# Catechol-O-Methyltransferase Val158Met Polymorphism on Striatum Structural Covariance Networks in Alzheimer’s Disease

**DOI:** 10.1007/s12035-017-0668-2

**Published:** 2017-07-13

**Authors:** Chiung-Chih Chang, Shih-Jen Tsai, Nai-Ching Chen, Chi-Wei Huang, Shih-Wei Hsu, Ya-Ting Chang, Mu-En Liu, Wen-Neng Chang, Wan-Chen Tsai, Chen-Chang Lee

**Affiliations:** 1 Department of Neurology, Cognition and Aging Center, Kaohsiung Chang Gung Memorial Hospital, Chang Gung University College of Medicine, #123, Ta-Pei Road, Niaosung, Kaohsiung, 833 Taiwan; 2 Psychiatric Department, Taipei Veterans General Hospital, Taipei, Taiwan; 3 Psychiatric Division, School of Medicine, National Yang-Ming University, Taipei, Taiwan; 4 Department of Radiology, Kaohsiung Chang Gung Memorial Hospital, Chang Gung University College of Medicine, Kaohsiung, Taiwan

**Keywords:** Alzheimer’s disease, Anatomical structural covariance, Default mode network, Genetic effect, Striatal network, Posterior cingulate cortex

## Abstract

The catechol-O-methyltransferase enzyme metabolizes dopamine in the prefrontal axis, and its genetic polymorphism (rs4680; Val158Met) is a known determinant of dopamine signaling. In this study, we investigated the possible structural covariance networks that may be modulated by this functional polymorphism in patients with Alzheimer’s disease. Structural covariance networks were constructed by 3D T1 magnetic resonance imaging. The patients were divided into two groups: Met-carriers (*n* = 91) and Val-homozygotes (*n* = 101). Seed-based analysis was performed focusing on triple-network models and six striatal networks. Neurobehavioral scores served as the major outcome factors. The role of seed or peak cluster volumes, or a covariance strength showing Met-carriers > Val-homozygotes were tested for the effect on dopamine. Clinically, the Met-carriers had higher mental manipulation and hallucination scores than the Val-homozygotes. The volume-score correlations suggested the significance of the putaminal seed in the Met-carriers and caudate seed in the Val-homozygotes. Only the dorsal-rostral and dorsal-caudal putamen interconnected peak clusters showed covariance strength interactions (Met-carriers > Val-homozygotes), and the peak clusters also correlated with the neurobehavioral scores. Although the triple-network model is important for a diagnosis of Alzheimer’s disease, our results validated the role of the dorsal-putaminal-anchored network by the catechol-O-methyltransferase Val158Met polymorphism in predicting the severity of cognitive and behavior in subjects with Alzheimer’s disease.

**Electronic supplementary material:**

The online version of this article (doi:10.1007/s12035-017-0668-2) contains supplementary material, which is available to authorized users.

## Introduction

Dopamine pathways modulate learning, memory, and neuropsychiatric presentations, and the catechol-O-methyltransferase (COMT) gene has been implicated in the enzymatic degradation of dopamine. In humans, a functional single nucleotide polymorphism (rs4680 G to A), consisting of a change in the coding exon at position 158 (Val158Met), has been reported to result in a two- to fourfold decrease in the activity of the COMT enzyme. Consequently, the low enzyme activity in Met/Met homozygotes results in increased dopamine levels that may preferentially affect prefrontal-related tasks [1].

Although the hypothesis of dopamine dysfunction in the early stage of Alzheimer’s disease (AD) is still under debate, several experimental models support the role of Aβ oligomers and dopamine dysfunction [2]. In addition, a recent meta-analysis suggested that COMT Val158Met Val/Val alleles were associated with an increased risk of AD in Asians [3]. In Taiwan, Wang et al. [4] found that the Val/Val genotype and apolipoprotein E4 (ApoE4) allele exert a synergistic effect on the risk of AD. An association of the rs4680 polymorphism with susceptibility to AD through a synergistic effect with ApoE4 alleles has also been reported in Caucasian [5] and Basque populations [6]. In AD, the COMT genotype has been shown to play a major role in the presentation of psychosis [7] and cognitive profiles [8]. Other studies, however, suggest no direct link between Val158Met and susceptibility to AD in the general population [3, 5, 9]. In Akil et al.’s study, pathological specimens of normal human brains [10] from individuals with the Val/Val genotype may have had higher levels of thyroxylase messenger RNA (mRNA) in mesencephalic dopamine neuronal populations projecting to the striatum. However, whether the higher levels of striatal dopamine can compensate for the lower dopamine concentration in the prefrontal cortex is unclear, and the mechanism by which the presentations are related to distinct network alterations remains to be explored.

The dopamine pathway represents one of the major biochemical signals in the striatum. Classically regarded as a motor structure, the striatum subserves a wide range of functions including cognitive, motivational, and emotional processes. In recent years, researchers have started to conceptualize the functional connectivity of distinct neural circuits associated with different sub-regions of the striatum. For instance, the reward-related function has been attributed to the ventral versus superior striatum [11]. Similarly, executive dysfunction has been reported in patients with putamen and caudate damage [12, 13]. Changes in the anatomical connections within the fronto-striatal circuits are related to syndrome complexes in the neurodegenerative spectrum. In AD, there may be lesser striatal atrophy, but the AD neuroimaging initiate group reported that the striatum may be an adjunctive biomarker [14]. Therefore, it is important to understand whether the psychiatric presentation [7] and cognitive profile [8] are related to COMT genotype-driven striatal pathways.

In 2006, Postuma and Dagher [15] proposed a seed-based model defining six striatal sub-regions and related cortical connections in the Talairach space. The striatal model divides the striatum into motor, associative, and limbic divisions. For the caudate nucleus, the most ventral to dorsal gradient spirals modulate the emotional/motivational aspects, followed by decision-making/executive control, and motor control functions [16, 17]. For the putamen, the functional connectivity has also been reported to show a rostral/caudal distinction that is primarily connected to the primary and secondary cortical motor areas and executive control [18]. Dopamine is necessary for prefrontal-dependent tasks, and the interest in COMT activity in neurodegenerative diseases has been based on its role in dopamine degradation [19]. Given that dopamine is a crucial mediator of neuronal function in AD, the striatal model [15] may be a good choice to understand the relationships between COMT Val158Met genotypes and neurobehavioral presentations.

Recent research has suggested that highly related regions show covariance in morphometric characteristics, the so-called structural covariance. With careful control of confounding factors, structural covariance networks (SCNs) have been used to test the influence of genotypes [20]. The triple-network model proposed by Menon [21] has been reported to be of great clinical relevance in AD and includes the default mode network (DMN) [22–24], salience network [25], and executive control network [26, 27]. Within the DMN, two subsystems are particularly relevant [28]: the “medial temporal lobe subsystem” and the “dorsal medial prefrontal cortex subsystem” (or the midline core subsystem).

Dopamine may affect cognition by facilitating neuronal synchrony; however, a direct correlation between COMT genotypes and prefrontal-striatal dopamine levels or gene-cognitive profiles remains controversial [10, 29]. The aims of the current study were to explore the network effects of COMT genotypes and to assess in vivo whether different genotype groups may modulate the SCN patterns and thereby determine neurobehavioral outcomes in AD. Based on a literature review, we selected the striatal model [15] to assess dopamine activity and the triple-network model [21] to assess which networks are characteristically affected in AD. Based on the biological properties of COMT on the prefrontal cortex, we hypothesized that the COMT Met158Val functional polymorphism may modulate selective striatal SCNs that determined the neurobehavioral scores in AD.

## Results

### Demographic Data, Cognitive Data, and NPI

The distribution of the genotypes of the Val158Met polymorphism in the patients with AD was in Hardy-Weinberg equilibrium. There were no significant differences in gender, age, and educational level between the two genotype groups (Table 1). In addition, there was no difference in ApoE4 status between the two groups. The Met-carriers had significantly higher scores in mental manipulation subdomains compared with the Val-homozygotes. The Met-carriers had higher scores in the hallucination domains.

**Table 1:** Demographical characteristics and neuropsychiatric tests in the COMT genotype groups in 192 Alzheimer’s disease

Genotype group	Met-carriers (*n* = 91)	Val/Val (*n* = 101)	*p* value
Age	73.3 ± 8.6	73.9 ± 7.2	0.62
Education (year)	7.3 ± 4.9	7.4 ± 4.9	0.91
Apolipoprotein E4 carrier (positive case, %)	32, 35.16%	34, 33.66%	0.88
Sex (male/female)	43/48	52/49	0.56
MMSE	19.9 ± 6.36	19.6 ± 6.81	0.772
Clinical dementia rating sum of box	3.6 ± 3.02	4.0 ± 3.22	0.375
Mental manipulation	*6.0* ± *3.30*	*4.7* ± *3.18*	*0.008*
Attention	6.3 ± 1.44	6.0 ± 1.63	0.202
Orientation	12.6 ± 5.23	12.5 ± 5.31	0.821
Long-term memory	8.2 ± 2.96	7.9 ± 2.72	0.497
Short-term memory	4.9 ± 3.87	5.6 ± 3.92	0.199
Abstract thinking	8.2 ± 2.91	7.9 ± 2.77	0.398
Drawing	7.7 ± 2.91	7.6 ± 3.06	0.797
Verbal fluency	5.1 ± 2.69	4.9 ± 2.87	0.663
Language	8.1 ± 2.20	7.9 ± 2.44	0.528
Executive function test	25.5 ± 8.17	23.5 ± 8.41	0.507
Total scores of CASI	67.2 ± 21.85	65.1 ± 22.26	0.510
Neuropsychiatric inventory scores total	8.49 ± 13.45	7.89 ± 9.99	0.723
Delusion	0.84 ± 2.32	0.54 ± 1.81	0.332
Hallucination	*0.31* ± *1.18*	*0.01* ± *0.10*	*0.012*
Aggression	0.53 ± 2.03	0.25 ± 1.03	0.223
Depression	1.15 ± 2.70	1.23 ± 2.59	0.847
Anxiety	0.35 ± 1.48	0.44 ± 1.48	0.695
Elation	0.08 ± 0.52	0.01 ± 0.10	0.207
Apathy	0.77 ± 2.50	0.88 ± 2.74	0.769
Disinhibition	0.11 ± 0.75	0.36 ± 1.75	0.216
Irritability	1.12 ± 2.41	0.98 ± 2.02	0.661
Aberrant motor behavior	0.42 ± 1.75	0.39 ± 1.73	0.901
Sleep disorders	2.45 ± 4.42	2.10 ± 4.12	0.569
Eating behavior	0.37 ± 1.28	0.71 ± 2.02	0.171

Data are presented as mean (standard deviation) or number (percentage; %); attention, verbal fluency, abstract thinking, and mental manipulation sub-domain scores of the CASI were added to assess executive function; APOE4 carriers were defined as the presence of one or two APOE4 alleles. The italicized word represents scores showing significance

*CASI* cognitive ability screening instrument, *COMT* catechol-O-methyltransferase

### Patterns of SCN and Genetic Variants

**Fig. 1:**
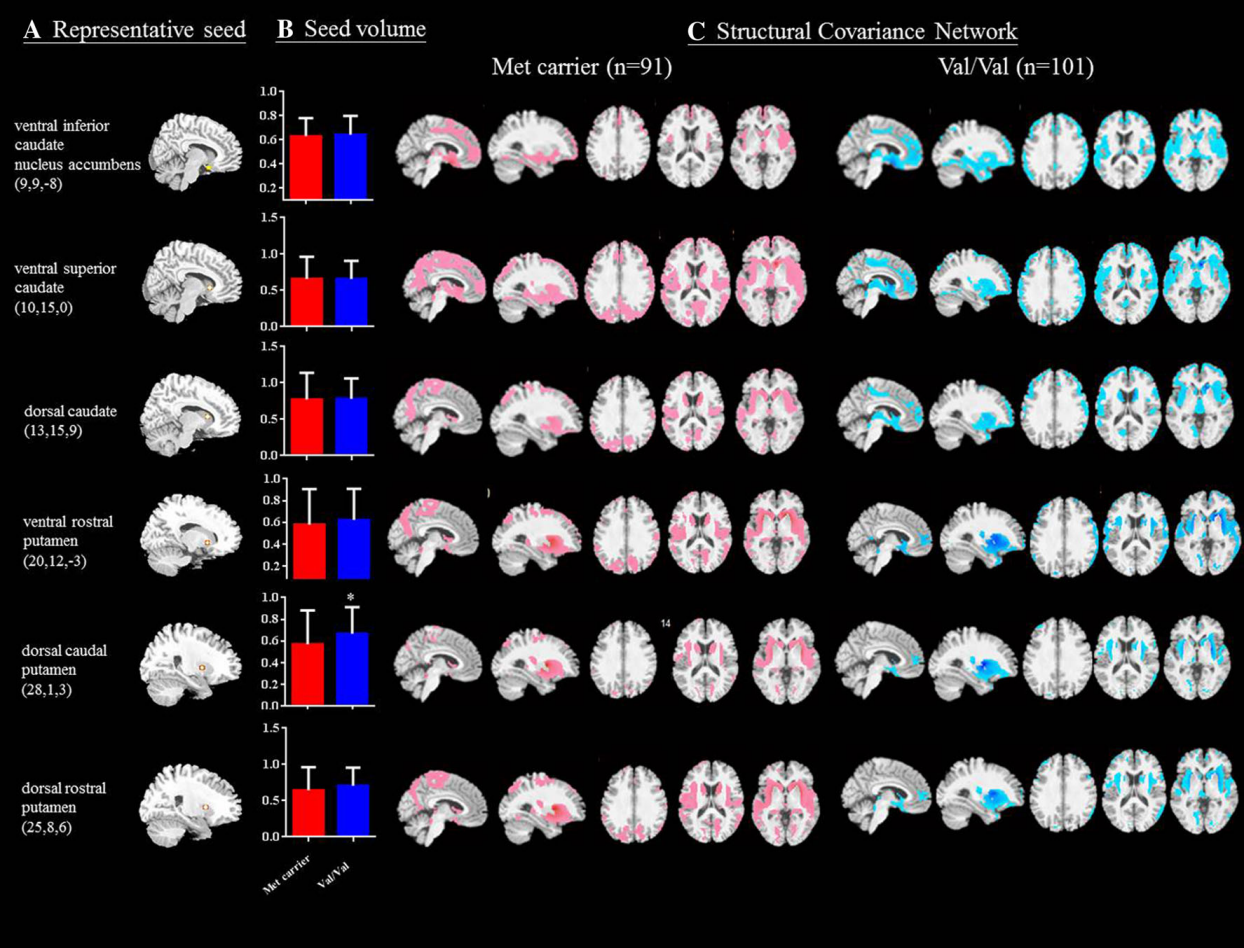
Statistical maps depicting brain areas in which the gray matter intensity covaried with **a** six target seeds, **b** seed volume comparisons, and **c** structural covariance networks (*Z*-statistic maps [*p* < 0.01, corrected with a false discovery rate with extended cluster voxels >100]) in all patients with Alzheimer’s disease with the catechol-O-methyltransferase Val158Met polymorphism (Met-carriers, *n* = 91; Val-homozygote carriers, *n* = 101). A significantly lower dorsal caudal putamen gray matter seed volume was found in the Met-carriers (*p* < 0.05). The images were displayed on a standard brain render

A direct comparison between the gray matter (GM) volume of the Met-carriers and Val-homozygotes using voxel-based morphometry [30] showed no significant differences (with the threshold set at *p* < 0.05, corrected for a false discovery rate (FDR) with a cluster size >100 voxels). In the striatal model, the dorsal caudal putamen (DCP) seed had a significantly higher volume in the Val-homozygotes (Fig. 1a), whereas in the triple-network model, the posterior cingulate cortex (PCC) seed volume was higher in the Met-carriers (Supplementary Figure 1B). The SCN patterns and related clusters in each genotype are shown in Fig. 1c, Supplementary 1C, and Supplementary Tables 1, 2, 3, 4, 5, 6, 7, 8, 9, 10, 11, 12, 13, 14, 15, 16, 17, 18, 19, 20.

### Peak Clusters Showing Significant Interactions Between Genotype Groups

**Fig. 2:**
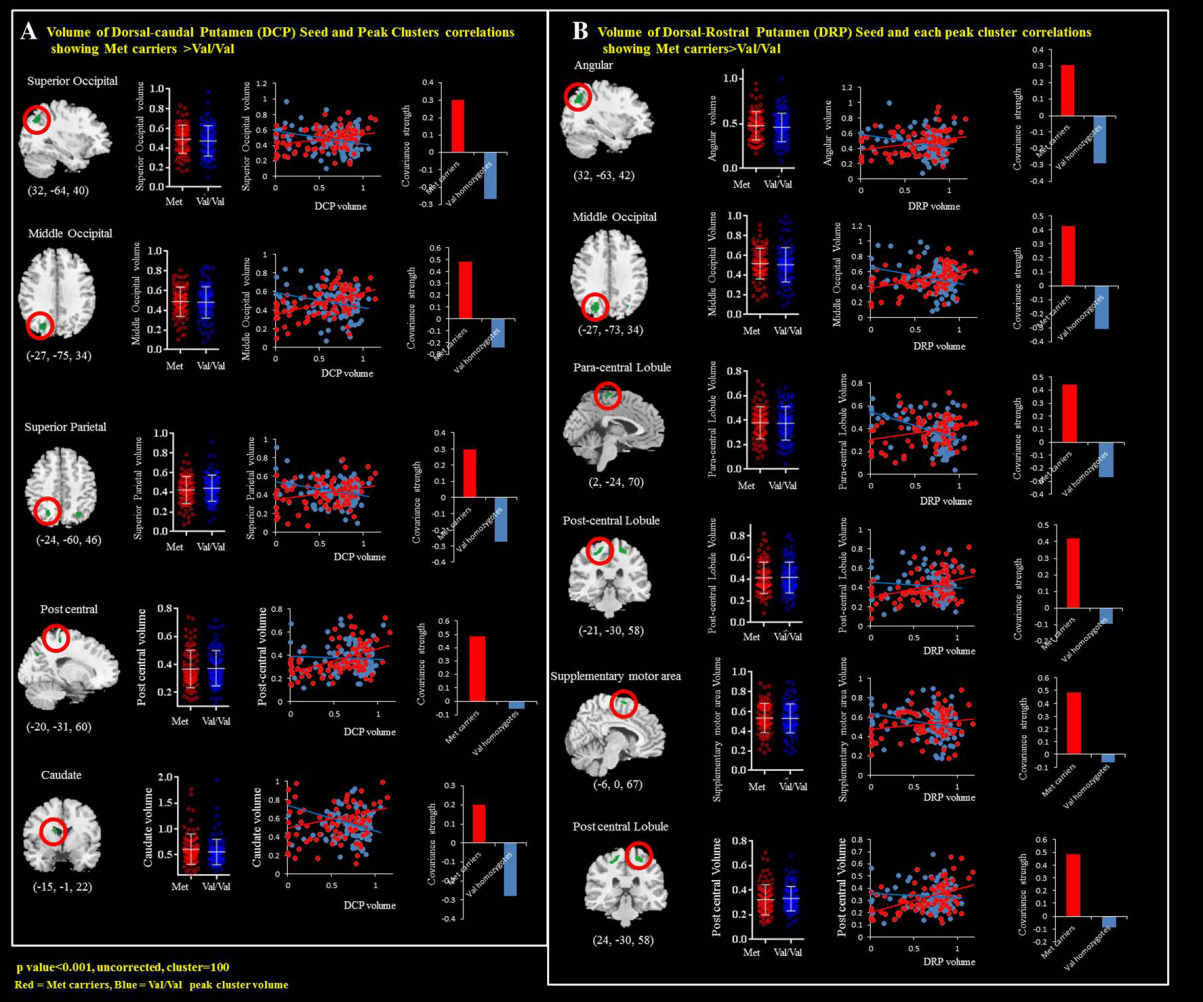
Peak clusters showing significant interactions of Met-carriers > Val-homozygotes from the **a** dorsal caudal putamen (DCP) and **b** dorsal rostral putamen (DRP) seed. There were five DCP-related peak clusters and six DRP-related clusters. *x*, *y*, *z* = Montreal Neurological Institute coordinates

For each seed, we explored the genotypic interactions with regard to the topography showing differences in structural covariance strength between seed and peak clusters (Fig. 2; supplementary Table 21; Supplementary Figure 1D). The left superior medial frontal region anchored to the entorhinal seed was the only significant cluster within the triple-network model showing Met-carriers > Val-homozygotes in covariance strength (Supplementary Figure 1D). In contrast, two seeds (Fig. 2a, DCP; Fig. 2b, dorsal rostral putamen (DRP)) within the striatal model exhibited 11 clusters showing Met-carriers > Val-homozygotes in covariance strength. However, there were no significant differences in direct comparisons of the volumes of the peak clusters between the two genotype groups.

### Relationships Between Seed Volume and Cognitive Score

We also explored whether the seed volumes were correlated with the cognitive test scores in each group (supplementary Table 22: triple-network model; Table 2: striatal model). For the triple-network model, only the posterior cingulate seed volume was significantly correlated with cognitive test scores in both genotypes, while more cognitive domains reached statistical significance in the Met-carriers. For the striatal model, the seed volumes showed variable correlations with the cognitive test scores and neuropsychiatric inventory (NPI) subdomains. As the DCP and DRP seed-connected striatal network showed greater Met-carriers > Val-homozygotes interactions, both seed volumes were related to the Mini-Mental State Examination (MMSE) scores in the Met-carriers and attention scores in the Val-homozygotes.

**Table 2:** Correlations between neurobehavioral test scores and seed volumes in the striatal model in each catechol-O-methyltransferase genotype groups

	Caudate						Putamen					
Seed no.	Ventral inferior		Ventral superior		Dorsal		Dorsal caudal		Dorsal rostral		Ventral rostral	
Genotype groups	Met-carriers	Val/Val	Met-carriers	Val/Val	Met-carriers	Val/Val	Met-carriers	Val/Val	Met-carriers	Val/Val	Met-carriers	Val/Val
MMSE	0.11	0.21*	0.20	0.23*	0.03	0.19	0.23*	0.14	0.23*	0.17	0.28*	0.25*
CASI total scores	0.11	0.20*	0.19	0.21*	0.02	0.18	0.21*	0.11	0.19	0.13	0.23*	0.22*
Executive function test	0.11	0.21*	0.19	0.21*	0.02	0.18	0.20	0.12	0.19	0.14	0.23*	0.21*
Mental Manipulation	−0.07	0.12	0.06	0.24*	−0.02	0.22*	0.15	0.14	0.15	0.16	0.13	0.22*
Attention	0.19	0.23*	0.27**	0.26**	0.12	0.14	0.17	0.23*	0.19	0.22*	0.23*	0.29**
Orientation	0.10	0.18	0.12	0.10	−0.04	0.05	0.09	0.10	0.08	0.09	0.15	0.14
Long-term memory	0.22*	−0.01	0.26*	0.13	0.14	0.12	0.29**	0.03	0.23*	0.07	0.24*	0.17
Short-term memory	0.11	0.29**	0.10	0.18	−0.14	0.15	0.08	0.10	0.03	0.09	0.11	0.15
Abstract thinking	0.09	0.18	0.15	0.10	−0.01	0.11	0.15	0.03	0.14	0.03	0.17	0.03
Drawing	−0.03	0.13	0.13	0.15	0.10	0.18	0.20	0.04	0.20	0.12	0.25*	0.16
Verbal fluency	0.11	0.14	0.22*	0.20*	0.06	0.12	0.22	0.08	0.24*	0.09	0.23*	0.17
Language	0.12	0.18	0.16	0.25*	0.11	0.23*	0.20	0.13	0.19	0.17	0.20	0.25*
NPI total scores	−0.04	0.06	0.02	−0.05	0.08	−0.01	−0.05	−0.03	0.00	−0.04	−0.03	0.02
Delusion	−0.14	−0.06	0.00	0.00	0.00	0.08	−0.08	0.06	−0.06	0.07	−0.07	0.04
Hallucination	−0.05	−0.14	−0.07	−0.21*	−0.12	−0.20*	−0.16	−0.27**	−0.16	−0.28**	−0.13	−0.22*
Aggression	−0.16	−0.04	−0.10	−0.02	−0.02	0.01	−0.09	−0.15	−0.03	−0.16	−0.12	−0.08
Depression	0.10	0.16	0.07	0.00	0.06	0.13	0.08	0.14	0.05	0.14	0.01	0.22*
Anxiety	0.04	0.21*	0.05	−0.01	0.00	0.08	0.05	0.02	0.00	0.09	−0.03	0.10
Elation	−0.09	−0.12	−0.17	−0.15	−0.04	−0.04	−0.16	−0.04	−0.04	−0.01	−0.11	−0.02
Apathy	−0.06	−0.22*	−0.10	−0.02	0.01	−0.07	−0.16	0.10	−0.09	0.03	−0.12	0.07
Disinhibition	−0.20	−0.02	−0.02	−0.12	−0.01	−0.03	−0.06	−0.05	−0.01	−0.08	0.01	−0.05
Irritability	−0.07	0.23*	0.02	0.11	0.00	0.07	−0.05	−0.09	−0.04	0.02	−0.06	0.13
Aberrant motor behavior	−0.14	−0.11	−0.07	−0.10	−0.05	−0.08	−0.20	−0.02	−0.15	−0.04	−0.15	−0.02
Sleep disorder	0.12	0.15	0.13	0.00	0.23*	0.02	0.16	−0.01	0.21*	−0.02	0.22*	−0.07
Eating behavior	0.04	−0.07	0.09	−0.09	0.06	−0.24*	−0.03	−0.27**	−0.06	−0.31**	0.03	−0.24*

*MMSE* Mini-Mental State Examination, *NPI* neuropsychiatric inventory, *CASI* cognitive ability screening instrument

Numbers indicate Pearson’s correlation coefficients—*p < 0.05; **p < 0.01

### Clinical Significance of Peak Clusters Showing Genotype Differences

The clinical significance of the aforementioned 12 peak clusters (Supplementary Figure 1D, Fig. 2) showing genotype interactions was evaluated by correlation analysis with cognitive tests (Table 3 for the Met-carriers, Table 4 for the Val-homozygotes). The results suggested more significant correlations with behavioral domains in the Met-carriers (Table 3) compared with the Val-homozygotes (Table 4).

**Table 3:** Significant correlations between peak cluster volumes and neurobehavioral test scores in the COMT Met-carriers

Seed region	PCC	Dorsal caudal putamen					Dorsal rostral putamen					
Peak cluster	Superior medial frontal	Superior occipital	Middle occipital	Superior parietal	Postcentral	Caudate	Angular	Middle occipital	Paracentral lobule	Caudate	SMA	Postcentral
Mini-Mental State Examination	0.15	0.16	0.42**	0.18	0.27*	−0.14	0.20	0.43**	−0.06	0.22*	0.03	0.25*
CASI total scores	0.11	0.19	0.45**	0.19	0.24*	−0.14	0.24*	0.47**	−0.07	0.19	−0.01	0.20
CASI EFT scores	0.11	0.20	0.42**	0.16	0.21*	−0.14	0.26*	0.42**	−0.07	0.20	0.02	0.17
Short-term memory	0.14	0.00	0.26*	0.12	0.13	−0.27*	0.01	0.30**	−0.17	0.08	−0.08	0.04
Orientation	0.09	0.20	0.41**	0.21*	0.19	−0.10	0.25*	0.43**	−0.03	0.11	−0.06	0.18
Long-term memory	0.06	0.21*	0.48**	0.20	0.26*	−0.04	0.25*	0.48**	0.01	0.22*	0.04	0.25*
Language	0.09	0.08	0.37**	0.12	0.26*	−0.11	0.10	0.38**	0.01	0.21*	−0.02	0.21
Drawing	0.13	0.19	0.32**	0.10	0.21*	0.00	0.22*	0.35**	−0.09	0.17	0.08	0.24*
Attention	0.11	0.23*	0.36**	0.17	0.22*	−0.03	0.23*	0.35**	0.13	0.22*	0.15	0.20
Verbal fluency	0.01	0.13	0.29*	0.13	0.11	−0.13	0.16	0.25*	−0.05	0.16	−0.05	0.06
Abstract thinking	0.08	0.17	0.41**	0.12	0.21	−0.16	0.24*	0.44**	−0.02	0.23*	−0.01	0.14
Mental manipulation	0.07	0.15	0.31*	0.10	0.17	−0.09	0.21*	0.31**	−0.17	0.07	0.03	0.16
Hallucination	−0.04	−0.15	−0.20	0.00	−0.07	0.00	−0.16	−0.20	0.26*	−0.07	0.15	−0.13
Anxiety	0–0.134	−0.02	0.21*	0.15	−0.03	−0.06	−0.05	0.19	0.04	−0.01	−0.10	−0.09
Elation	−0.067	−0.17	−0.20	0.10	−0.10	0.03	−0.17	−0.24*	0.03	−0.11	0.02	−0.10
Apathy	−0.087	−0.23*	−0.29**	−0.06	−0.17	−0.03	−0.21*	−0.29**	0.14	−0.14	0.04	−0.04
Aberrant motor behavior	−0.129	−0.13	−0.20	0.01	−0.22*	−0.04	−0.12	−0.23*	0.03	−0.22*	−0.04	−0.14
Eating behavior	−0.073	−0.13	−0.06	0.11	0.09	0.21*	−0.20	−0.07	0.19	0.09	0.10	−0.02

Selected peak clusters indicate structural covariance strength showing Met-carriers > Val-homozygotes

*PCC* posterior cingulate cortex, *SMA* supplementary motor area, *COMT* catechol-O-methytransferase

Numbers indicate Pearson’s correlation coefficients—*p < 0.05; **p < 0.01

**Table 4:** Significant correlations between peak cluster volume and neurobehavioral test scores in the Valine homozygotes

Seed	PCC	DCP seed					DRP seed					
Peak cluster	Superior medial frontal	Superior occipital	Middle occipital	Superior parietal	Postcentral	Caudate	Angular	Middle occipital	Paracentral lobule	Caudate	SMA	Postcentral
CASI EFT scores	−0.05	0.15	0.12	0.20*	0.09	−0.05	0.05	0.08	−0.12	0.13	−0.05	0.05
Abstract thinking	0.10	0.19	0.05	0.15	0.15	−0.10	0.07	0.03	−0.10	0.20*	0.07	0.13
Hallucination	0.04	0.02	0.06	0.19**	0.11	−0.05	−0.03	0.09	0.21*	0.05	0.00	−0.13
Aggression	0.18	0.20*	0.20*	−0.01	0.06	0.12	0.24*	0.26**	0.13	0.00	0.25*	0.08
Depression	0.11	0.09	0.04	0.03	0.20*	−0.03	0.16	0.04	0.06	0.17	0.12	0.27**
Elation	−0.02	−0.24*	−0.26**	−0.12	−0.06	−0.07	−0.24*	−0.24*	−0.17	−0.06	−0.12	−0.10

The *CASI* seed regions are dorsal rostral putamen (DRP) and dorsal caudal putamen (DCP)

*MMSE* Mini-Mental State Examination, *EFT* executive function test, *CASI* cognitive ability screening instrument, *PCC* posterior cingulate cortex, *SMA* supplementary motor area

Numbers indicate Pearson’s correlation coefficients—*p < 0.05; **p < 0.01

## Discussion

This study provides data on the neurobehavioral and network influence of COMT Val158Met in patients with AD. The findings can be considered in three levels: clinical, cortical regional, and network level. From the clinical level, the neurobehavioral comparisons between the Met-carriers and Val-homozygotes showed that the lower COMT activity group had higher scores in mental manipulation scores and hallucination domains. From the regional aspect, the significant correlations between test scores and the seed or peak cluster volumes demonstrated the clinical significance of PCC in DMN and all six striatum regions. Lastly, although the triple-network model has been well studied in AD, our network analysis results support a higher weighting of striatum-related circuits according to the COMT genotype, of which the DCP- or DRP-interconnected networks that contributed differently to the prediction of clinical outcome were most pronounced.

### COMT Genotypes in AD Symptomatology Modulation

Although the disease-causing genetic profiles for AD have been identified in genome-wide association studies, these genetic markers have not been fully investigated with regard to outcome correlations. This formed the basis of the current study. Met-carriers can be considered as a group with long-term lower COMT activity compared to Val-homozygotes. Our Met-carriers presented with higher mental manipulation test and hallucination scores, supporting the biological link between the COMT Val158Met polymorphism and prefrontal dopamine metabolism [31]. Our results also validate those reported in normal elderly [32] and in patients with dementia [8] in that those with low COMT enzyme activity perform better in prefrontal-directed tasks or that it is related to psychiatric manifestations [7, 33]. Of note, COMT activity can be confounded by physiological factors such as gender, age, sex hormones, and ApoE4 status [4, 34]. However, the association between COMT polymorphisms and decline in executive control with aging is controversial [35, 36].

The clinical correlation suggests that the PCC volume can be used to predict cognitive but not NPI performance (Supplementary Table 22). The peak clusters anchored by the PCC and the clinical correlations were also not significant in the Met-carriers (Table 3) or Val/Val group (Table 4). Therefore, the COMT Val158Met polymorphism showed greater weighting in the striatal network than in the triple network to predict cognitive symptoms.

### COMT Genotype Effects on the Clinical Presentations Modulated by Large-Scale Striatum Networks

The DCP and DRP seed volumes were related to the MMSE and long-term memory scores in our Met-carriers, whereas significant correlations between the DCP and DRP seed volumes were found in the attention scores, hallucination, and eating behavior in the Val-homozygotes. There are several possible mechanisms as to how the genotype may modulate the neurobehavioral profile. Such differences in clinical profile may reflect the genotype modulation associated with COMT activity. In addition, within the DCP- and DRP-interconnected peak clusters, the clusters and seeds each contribute differently to the genotype group, leading to different levels of COMT enzymatic expression that affect anatomical correlations.

The peak clusters showing interactions with covariance strength between the genotype groups (i.e., Met-carriers > Val-homozygotes) were tested for their effect on dopamine. As more statistically significant neurobehavioral domains were found in the Met-carriers in the network showing higher covariance strength (Table 3: Met-carriers; Table 4: Val-homozygotes), the mechanisms as to why an increased dopamine expression may be related to better cognitive performance and more psychiatric presentations were validated in the striatum network.

### COMT Genotypes Modulated the Dorsal Putaminal Dopaminergic Network

In our analysis, the Val/Val group had higher dorsal caudal putamen volumes (Fig. 1b) but lower mental manipulation and hallucination scores (Table 1), suggesting that the dorsal caudal putamen was responsible for impaired cognitive modulation but a lower tendency of psychiatric symptoms. Based on the correlation analysis (Table 2), a significant inverse correlation with hallucination score was shown in the Val/Val group, suggesting that the integrity of the dorsal putamen may protect against hallucinations in AD.

Our network analysis further supported the genetic effects of COMT on the striatum and its functional circuits. Based on the interactions, the dorsal putaminal networks were more important than the ventral networks. In the dorsal putaminal networks, correlation analysis showed different patterns between DCP or DRP and neurobehavioral patterns. These network differences may reflect differences in genotype groups and neuroanatomy, as greater connections were found between DCP and BA 6 and between DRP and dorsal ACC [37].

The ventral rostral putamen seed along with the rostral portion of the anterior cingulate cortex and dorsolateral prefrontal cortex was associated with conflict monitoring and error-related processes. Genotype interactions in the ventral rostral putamen seed-connected clusters were lacking. Nonetheless, the significant correlations between ventral rostral putamen seed volume and MMSE, cognitive ability screening instrument (CASI) total score, executive function test, and attention scores in both genotype groups suggest the clinical role of the ventral rostral putamen seed.

### Caudate Seed and Clinical Features in the Val-Homozygotes

None of the peak clusters connected to the ventral striatum seed showed greater structural covariance strength in the Met-carriers. This may be due to a minor genotype modulation effect on the ventral striatum-interconnected clusters. The correlations between ventral striatum seed and neurobehavioral symptoms were still significant and displayed a parallel correlation pattern in the superior ventral striatum or inferior ventral striatum, especially in the Val-homozygotes. These results may be related to the identical structural projection zone of the superior ventral striatum [38–41] and inferior ventral striatum seed [42–45].

For the dorsal caudate nucleus, the anatomical connection has been associated with the dorsolateral prefrontal cortex and executive control regions [46–48]. The dorsal caudate nucleus seed volume was related to the mental manipulation and language ability in the Val-homozygotes. Of note, in the Val-homozygotes, the COMT genotype effect that significantly modulated the caudate seed determined the clinical features.

### Inconsistent Effect of COMT on Cortices and Possible Explanations

Individuals with the Val/Val genotype have been reported to have higher levels of thyroxylase mRNA in mesencephalic dopamine neuronal populations that project to the striatum [10], which may explain why the COMT valine allele leads to susceptibility to psychosis. However, as the pathological specimens in Akil et al.’s study excluded those from patients with AD, the direct application of their results is not possible. In our analysis, the Val/Val group had higher dorsal caudal putamen volumes (Fig. 1b) but lower mental manipulation and hallucination scores (Table 1), suggesting that the dorsal caudal putamen is responsible for impaired cognitive modulation but a lower tendency toward psychiatric symptoms. In our correlation analysis (Table 2), a significant inverse correlation with hallucination score was shown in the Val/Val group, suggesting that the integrity of the dorsal putamen may protect against hallucinations in AD.

In addition to the physiological role of striatal networks, several factors in AD may also contribute to the inconsistent effect of COMT. For example, the genetic expression of COMT and the effect of the dopamine system can be influenced by aging, amyloid load, and disease severity. During the physiological aging process, decreased dopamine release, decreased receptor expression (especially D2), and reduced transporter expression are found in the caudate, putamen, hippocampus, and prefrontal cortex of human brains [49, 50]. In AD, atrophy of the caudate [14] and putamen [51] has also been reported, and decreases in volume have also been correlated with cognitive deficits. In addition, the integrity of nicotinic acetylcholine receptors can be affected by amyloid-related pathologies in AD [52, 53] such as neuronal homeostasis, synaptic plasticity, learning, and memory.

The striatum network in AD is mediated by different pathways and neurotransmitters, and dopaminergic pathways such as nigrostriatal pathways (substantia nigra and striatum) modulate voluntary movement and mesocorticolimbic pathways (ventral tegmental area, hippocampus, nucleus accumbens) modulate cognitive-behavior-reward function. Although dopamine levels have been reported to be higher in the striatum of individuals with the Val/Val genotype than in those with the Val/Met genotype [10], the areas showing most significant differences were in the ventral tier of the substantia nigra. Animal studies have shown that diminished prefrontal dopamine neurotransmission leads to upregulation of striatal dopamine activity, while higher dopamine levels in Val/Val may downregulate the activity at the level of the prefrontal cortex and also the mesolimbic system. The increased mesencephalic dopamine activity with the Val allele may regulate cortical glutaminergic projections (prefrontal, hippocampus, and amygdala) back to the mesolimbic pathways. The dynamic changes in network alterations along with the pathological cascades may have confounded the data with regard to the effects of the COMT genotype.

### Study Limitations

An important limitation of this study is that we did not include a control group. The enrolment of controls may have helped to elucidate whether the COMT polymorphism has a similar effect on the normative brain network. Our results support published data on elderly healthy subjects that genetic variations of the COMT polymorphism may mediate pre-frontal-related tasks [31]. However, direct analysis of SCN patterns in the striatal or triple network with changes in structural covariance strength in controls was not available. The results of this suggest how the COMT genetic polymorphism may interfere with structural networks and may be correlated with the neurobehavioral symptoms in AD. Another potential limitation is that we reported the peak clusters which showed greater covariance in the Met-carriers compared with the Val-homozygotes. Such group stratification only explores the intra-cerebral long-term effects of dopamine on the neurobehavioral outcomes. The expression of the COMT genotype has been reported to be affected by gene-environment interactions [54] which could not be fully included in this study model. Nonetheless, our results may suggest that the underlying sensitivity of genotype groups or the dopamine transmitter system is due to an environmental impact. Third, it has also been reported that cross-sectional findings of genetic effects could not be replicated in longitudinal observations [35, 36]. Further longitudinal studies including more extensive cognitive test items are warranted.

## Conclusion

In AD, the COMT Val158Met polymorphism modulates the striatal network rather than the triple network with regard to predicting symptoms. The genotype group itself, seed volume, or striatal network provided variable predictions of the clinical features. In the striatal network, greater covariance strength in the Met-carriers was found in the DCP- and DRP-interconnected networks that were suggestive of a long-term dopamine-related effect. Along with the significant clinical correlations, the DCP- and DRP-interconnected networks may be considered to be the major networks modulated by the COMT genotype.

## Materials and Methods

This study was conducted in accordance with the Declaration of Helsinki and was approved by the Institutional Review Board of Chang Gung Memorial Hospital. The study participants were treated at the Cognition and Aging Center, Department of General Neurology, Kaohsiung Chang Gung Memorial Hospital. A total of 192 subjects (95 males, 97 females) were included after the consensus of a panel composed of neurologists, neuropsychologists, neuroradiologists, and experts in nuclear medicine [55]. AD was diagnosed according to the International Working Group criteria [45] with a clinical diagnosis of typical AD. All of the patients were in a stable condition under acetylcholine esterase inhibitor treatment from the time of diagnosis. The exclusion criteria were a past history of clinical stroke, a modified Hachinski ischemic score >4, and depression.

### Study Working Scheme

Because of the limited number of subjects in the Met/Met group, we grouped the Val/Met and Met/Met subjects into the Met-carrier group in all subsequent analysis: Met-carriers (Met/Met = 20, Met/Val = 71, *n* = 91) and Val-homozygotes (*n* = 101). The working scheme was as follows. First, the SCNs were established by seed-based correlation analysis. Differences in each seed regional volume were compared between two genotype groups and correlated with the neurobehavioral scores. In order to evaluate the dopaminergic network effects, only the peak clusters showing Met-carriers > Val/Val in covariance strength were considered as statistically significant. The volumes of the significant peak clusters were selected and correlated with cognitive test scores to evaluate the clinical relevance in each genotype group.

### Clinical and Neurobehavioral Assessments

After enrolment, demographic data of each patient were recorded. A trained neuropsychologist administered the neurobehavioral tests. The MMSE scores and CASI total scores were used as a global assessment of cognitive function. Attention, verbal fluency, abstract thinking, and mental manipulation sub-domain scores of the CASI were used to assess executive function test (EFT) [56], while the non-executive domains included orientation, short- and long-term memory, language ability, and drawing. For the behavioral observations, we used the 12-item version of the NPI [57], with scores ranging from 0 to 144.

### Genotyping for COMT

Genotyping of COMT Val158Met was performed using the polymerase chain reaction-restriction fragment length polymorphism method. In brief, a DNA fragment containing the Val/Met polymorphism in COMT was amplified by polymerase chain reaction with primers reported by Lachman et al. [58]. The Val/Met polymorphism was differentiated by the *Nla*III restriction fragment length polymorphism analyzed on 10% polyacrylamide gel. Partial digestion and contamination amplification were ruled out by the complete digestion of an intrinsic restriction site and a blank sample in each batch of experiments, respectively. The ApoE genotype was determined using a PCR-restriction fragment length polymorphism assay and restriction enzyme *Hha*I. ApoE4 carriers were defined as those with one or two E4 alleles.

### Image Acquisition

MR images were acquired using a 3.0T MRI scanner (Excite, GE Medical Systems, Milwaukee, WI, USA). Structural images were acquired for SCN constructions using the following protocols: a T1-weighted, inversion-recovery-prepared, three-dimensional, gradient-recalled acquisition in a steady-state sequence with a repetition time/echo time/inversion time of 8600 ms/minimal/450 ms, a 256 × 256 mm field of view, and a 1-mm slice sagittal thickness with a resolution of 0.5 × 0.5 × 1 mm^3^.

### Data Analysis for Neuroimaging Biomarkers

Image preprocessing and statistical analysis were performed using SPM8 (SPM8, Wellcome Trust Centre of Cognitive Neurology, University College London, UK, http://www.fil.ion.ucl.ac.uk/spm/). The T1 images were reoriented, realigned, and normalized using the standard Montreal Neurological Institute space. The images were then segmented into GM and white matter. Related tissue segments were used to create a custom template using the diffeomorphic anatomical registration using exponentiated lie algebra (DARTEL) approach. The DARTEL approach is one of the highest ranking registration methods in patients with AD [59]. The modulated and warped images were then smoothed using a Gaussian kernel of 8 mm full width at half maximum.

### Images Analysis

To investigate the SCNs, 10 regions of interest, representing seeds, were selected from the 192 preprocessed images. The striatal network [15] included the following seeds (Fig. 1a): inferior ventral striatum [coordinates: [9, 9, −8], superior ventral striatum [coordinates: 10, 15, 0]; dorsal caudate [coordinates: 13, 15, 9]; ventral rostral putamen [coordinates: 20, 12, −3]; dorsal caudal putamen [DCP; coordinates: 28, 1, 3]; dorsal rostral putamen [DRP; coordinates: 25, 8, 6]. The coordinates of seed in the triple-network model included the right entorhinal cortex [coordinates: 25, −9, −28] and left posterior cingulate cortex [PCC; coordinates: −2, −36, 35] of the DMN, right frontoinsular cortex [coordinates: 38, 26, −10] of the salience network, and right dorsolateral prefrontal cortex [coordinates: 44, 36, 20] of the executive control network [25] (supplementary fig 1).

From the modified GM images, the GM volumes of a 4-mm radius sphere around the seed coordinates were also calculated, followed by 10 separate correlation analyses using the extracted GM volumes as the covariates of interest, to form the SCN. The two genotype groups were modeled separately. Based on the equivalent sample sizes in each genotype group, T contrasts were used to identify voxels that showed positive correlations for each seed. The results reflected the SCNs anchored by each seed. The threshold was set at *p* < 0.01, corrected for false discovery rate (FDR) with a cluster size >100 voxels.

In addition, to investigate how genetic variance may interfere with SCN covariance strength, voxels showing significant differences in the regression slopes in each seed-peak cluster correlations were compared that pointed to interactions between Met-carriers > Val-homozygotes. Specific T contrasts were established to map the voxels that expressed significant between-group associations.

For the peak clusters showing significant between-group differences, a 4-mm radius sphere was placed on the peak voxel, and the GM volumes were then calculated. To evaluate the clinical significance of the seed or the identified peak voxel, we used correlation analysis with the cognitive test or NPI scores as outcome measures. The threshold was set at *p* < 0.05 with multiple corrections.

### Statistical Analysis

Clinical and laboratory data were expressed as mean ± standard deviation. The Student *t* test was used to compare the continuous variables and chi-square test for category variables. Pearson’s correlation was used to analyze the seed or cluster volume on predicting the cognitive or NPI scores. All statistical analyses were conducted using SPSS software (SPSS version 22 for Windows®, SPSS Inc., Chicago, IL). Statistical significance was set at *p* < 0.05.

## Electronic Supplementary Material

Supplementary Figure 1 Statistical maps depicting brain areas in which the gray matter intensity covaried with (**a**) four target seeds, (**b**) seed volume comparisons, and (**c**) structural covariance networks (Z-statistic maps [*p* < 0.01, corrected with a false discovery rate with extended cluster voxels >100]) in all patients with Alzheimer’s disease with the catechol-O-methyltransferase Val158Met polymorphism (Met-carriers, *n* = 91; Val-homozygotes carriers, *n* = 101). A significantly lower posterior cingulate cortex gray matter seed volume was found in the Met-carriers (*p* < 0.05). The images were displayed on a standard brain render. (GIF 455 kb)

High resolution image (TIFF 645 kb)

Supplementary Table 1 (DOCX 21 kb)

Supplementary Table 2 (DOCX 23 kb)

Supplementary Table 3 (DOCX 20 kb)

Supplementary Table 4 (DOCX 19 kb)

Supplementary Table 5 (DOCX 19 kb)

Supplementary Table 6 (DOCX 19 kb)

Supplementary Table 7 (DOCX 18 kb)

Supplementary Table 8 (DOCX 21 kb)

Supplementary Table 9 (DOCX 21 kb)

Supplementary Table 10 (DOCX 20 kb)

Supplementary Table 11 (DOCX 19 kb)

Supplementary Table 12 (DOCX 20 kb)

Supplementary Table 13 (DOCX 20 kb)

Supplementary Table 14 (DOCX 21 kb)

Supplementary Table 15 (DOCX 21 kb)

Supplementary Table 16 (DOCX 22 kb)

Supplementary Table 17 (DOCX 20 kb)

Supplementary Table 18 (DOCX 22 kb)

Supplementary Table 19 (DOCX 20 kb)

Supplementary Table 20 (DOCX 21 kb)

Supplementary Table 21 (DOCX 23 kb)

Supplementary Table 22 (DOCX 25 kb)
