# Supplementary figures and images for: Catechol-O-Methyltransferase Val158Met Polymorphism on Striatum Structural Covariance Networks in Alzheimer’s Disease
Source: Mol Neurobiol. 2017 Jul 13;55(6):4637–49. doi: 10.1007/s12035-017-0668-2 (PMC5948254; doi:10.1007/s12035-017-0668-2)

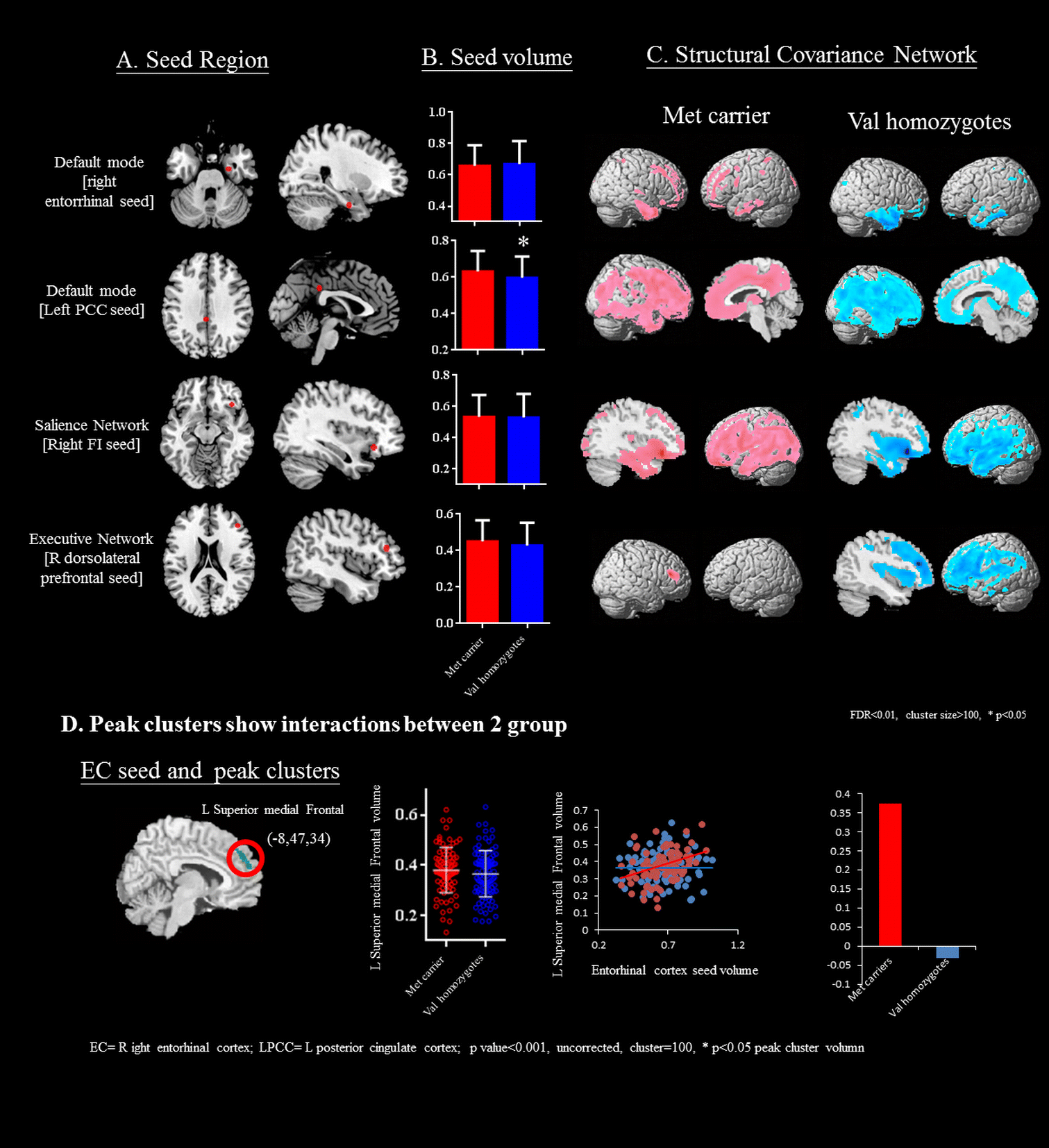

Supplement: Supplementary file 1 — Statistical maps depicting brain areas in which the gray matter intensity covaried with (a) four target seeds, (b) seed volume comparisons, and (c) structural covariance networks (Z-statistic maps [p < 0.01, corrected with a false discovery rate with extended cluster voxels >100]) in all patients with Alzheimer’s disease with the catechol-O-methyltransferase Val158Met polymorphism (Met-carriers, n = 91; Val-homozygotes carriers, n = 101). A significantly lower posterior cingulate cortex gray matter seed volume was found in the Met-carriers (p < 0.05). The images were displayed on a standard brain render. (GIF 455 kb) [file 12035_2017_668_Fig3_ESM.gif]

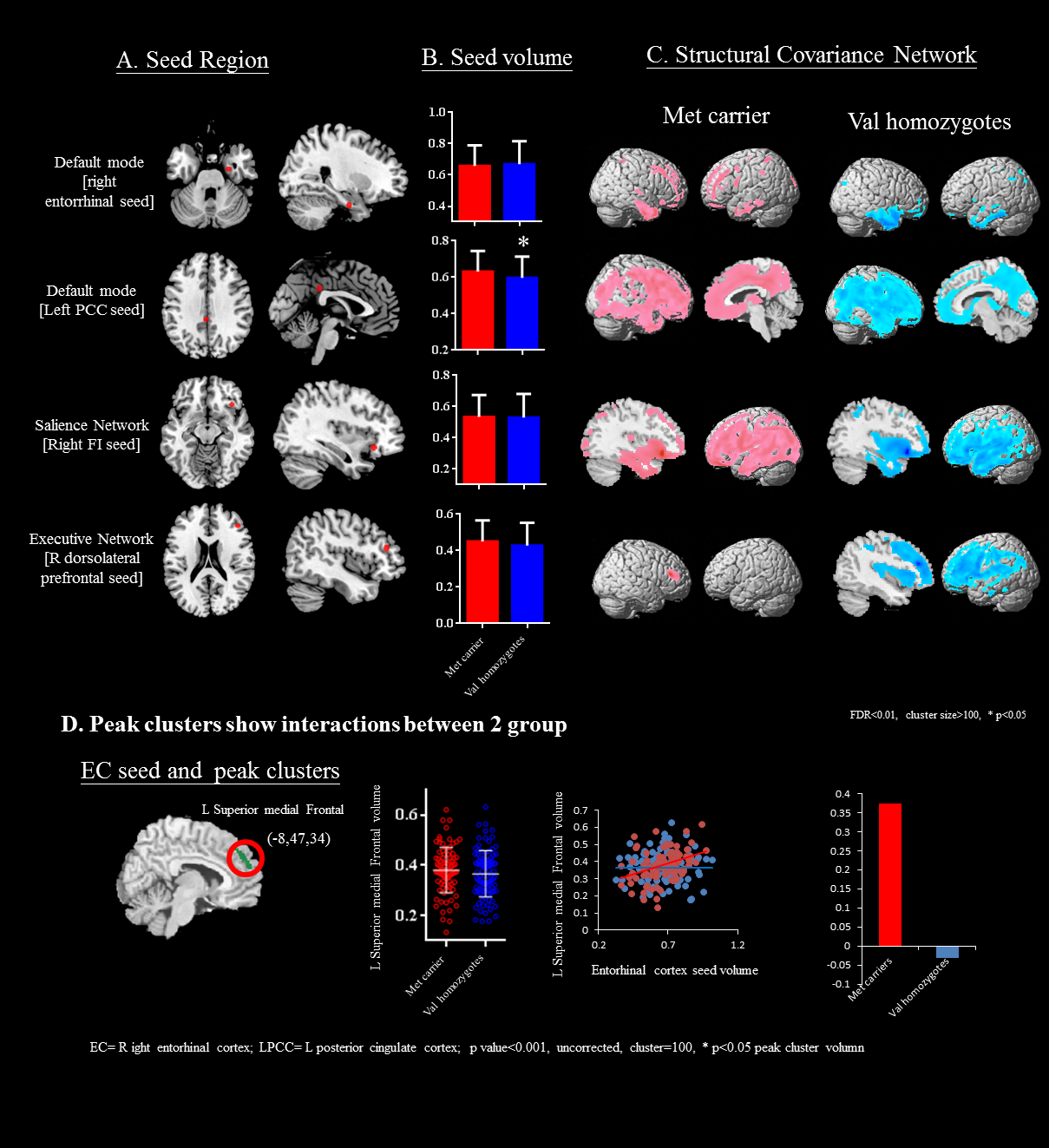

Supplement: Supplementary file 2 — High resolution image (TIFF 645 kb) [file 12035_2017_668_MOESM1_ESM.tif]
