# Supplementary material for: Catechol-O-Methyltransferase Val158Met Polymorphism on Striatum Structural Covariance Networks in Alzheimer’s Disease
Source: Mol Neurobiol. 2017 Jul 13;55(6):4637–49. doi: 10.1007/s12035-017-0668-2 (PMC5948254; doi:10.1007/s12035-017-0668-2)
Supplement: Supplementary file 3 — (DOCX 21 kb) [file 12035_2017_668_MOESM2_ESM.docx]

**Supplementary table 1. Structural covariance network for catechol-O-methyltransferase** **Met carrier with right entorhinal cortex as seed.**

| **Main Cluster** | **Peak regions** | **Side** | **Stereotaxic coordinates** | | | **Extent** | **Max T** | **P-value** |
| --- | --- | --- | --- | --- | --- | --- | --- | --- |
|  |  |  | x | y | z |  |  |  |
| ParaHippocampal |  | R | 26 | -12 | -29 | 4131 | 15.52 | <0.001 |
|  | Middle Temporal | R | 63 | -13 | -18 | s.c | 5.21 | <0.001 |
|  | Middle Temporal | R | 60 | -5 | -26 | s.c | 5.09 | <0.001 |
| ParaHippocampal |  | L | -24 | -18 | -26 | 1885 | 7.79 | <0.001 |
|  | Inferior Temporal | L | -48 | -10 | -33 | s.c | 5.32 | <0.001 |
|  | Middle Temporal Pole | L | -41 | 9 | -30 | s.c | 5.12 | <0.001 |
| Superior medial Frontal |  | L | -8 | 45 | 37 | 5433 | 7.17 | <0.001 |
|  | Superior medial Frontal | L | -8 | 56 | 21 | s.c | 7.11 | <0.001 |
|  | Middle Frontal | L | -29 | 38 | 25 | s.c | 5.54 | <0.001 |
| Rectus |  | R | 12 | 44 | -18 | 4356 | 6.21 | <0.001 |
|  | Superior medial Frontal | R | 11 | 44 | 37 | s.c | 6.12 | <0.001 |
|  | Superior medial Frontal | R | 14 | 50 | 30 | s.c | 5.67 | <0.001 |
| Postcentral |  | L | -29 | -45 | 57 | 368 | 4.94 | <0.001 |
| Inferior orbital Frontal |  | R | 18 | 17 | -20 | 300 | 4.9 | <0.001 |
|  | Inferior orbital Frontal | R | 33 | 26 | -17 | s.c | 4.45 | <0.001 |
| Superior Occipital |  | L | -18 | -99 | 18 | 229 | 4.75 | <0.001 |
|  | Cuneus | L | -9 | -93 | 28 | s.c | 4.68 | <0.001 |
|  | Middle Occipital | L | -26 | -96 | 18 | s.c | 3.51 | <0.001 |
| Postcentral |  | R | 27 | -43 | 57 | 313 | 4.71 | <0.001 |
|  | Superior Parietal | R | 21 | -52 | 59 | s.c | 3.97 | <0.001 |
| Frontal inferior operculum |  | L | -45 | 8 | 18 | 307 | 4.58 | <0.001 |
|  | Frontal inferior operculum | L | -42 | 5 | 27 | s.c | 4.37 | <0.001 |
| Precentral |  | L | -21 | -15 | 63 | 111 | 4.31 | <0.001 |
| Inferior Parietal |  | L | -54 | -40 | 37 | 133 | 4.19 | <0.001 |
| Precentral |  | L | -38 | 6 | 45 | 117 | 4.17 | <0.001 |
| Precuneus |  | L | -12 | -72 | 36 | 280 | 4.16 | <0.001 |
|  | Cuneus | L | -11 | -61 | 19 | s.c | 3.58 | <0.001 |
| Superior Temporal |  | R | 65 | -18 | 3 | 102 | 4.08 | <0.001 |
|  | Superior Temporal | R | 63 | -7 | -2 | s.c | 3.5 | <0.001 |

Peak regions are within the Main cluster

Max T is the maximum T statistic for each local maximum. P<0.05 based on non-stationary cluster-extent False discovery rate correction. s.c: same clusters
