# Supplementary material for: Catechol-O-Methyltransferase Val158Met Polymorphism on Striatum Structural Covariance Networks in Alzheimer’s Disease
Source: Mol Neurobiol. 2017 Jul 13;55(6):4637–49. doi: 10.1007/s12035-017-0668-2 (PMC5948254; doi:10.1007/s12035-017-0668-2)
Supplement: Supplementary file 4 — (DOCX 23 kb) [file 12035_2017_668_MOESM3_ESM.docx]

**Supplementary table 2.Structural covariance network for catechol-O-methyltransferase Valine homozygotes with right entorhinal cortex as seed**

| **Main Cluster** | **Peak regions** | **Side** | **Stereotaxic coordinates** | | | **Extent** | **Max T** | **P-value** |
| --- | --- | --- | --- | --- | --- | --- | --- | --- |
|  |  |  | x | y | z |  |  |  |
| ParaHippocampal |  | R | 27 | -9 | -29 | 12454 | 14.42 | <0.001 |
|  | Fusiform | R | 27 | -4 | -39 | s.c | 6.58 | <0.001 |
|  | Fusiform | R | 41 | -12 | -36 | s.c | 6.48 | <0.001 |
| ParaHippocampal |  | L | -24 | -10 | -27 | 6010 | 6.5 | <0.001 |
|  | Inferior Temporal | L | -41 | -28 | -26 | s.c | 6.45 | <0.001 |
|  | Inferior Temporal | L | -50 | -36 | -23 | s.c | 6.09 | <0.001 |
| Lingual |  | L | -11 | -33 | -3 | 258 | 4.63 | <0.001 |
| Precuneus |  | L | -11 | -70 | 57 | 470 | 4.52 | <0.001 |
|  | Superior Occipital | L | -20 | -81 | 37 | s.c | 4.07 | <0.001 |
| Olfactory |  | L | -2 | 20 | -18 | 1217 | 4.35 | <0.001 |
|  | Rectus | R | 8 | 57 | -18 | s.c | 3.92 | <0.001 |
|  | Frontal medial orbital | R | 9 | 62 | -11 | s.c | 3.88 | <0.001 |
| Lingual |  | L | -11 | -72 | -9 | 1312 | 4.31 | <0.001 |
|  | Lingual | L | -15 | -63 | 0 | s.c | 4.22 | <0.001 |
|  | Lingual | L | -6 | -67 | 3 | s.c | 4.14 | <0.001 |
| Lingual |  | R | 3 | -72 | -3 | 121 | 4.15 | <0.001 |
| Thalamus |  | R | 18 | -30 | 0 | 163 | 4.14 | <0.001 |
| Inferior Parietal |  | L | -39 | -54 | 42 | 148 | 4.11 | <0.001 |
| Superior Occipital |  | R | 24 | -79 | 34 | 164 | 4.03 | <0.001 |
| Superior Occipital |  | R | 23 | -70 | 40 | s.c | 3.85 | <0.001 |
| Middle Temporal |  | L | -56 | -27 | -8 | 106 | 3.85 | <0.001 |
| Middle Frontal |  | R | 29 | 56 | 4 | 106 | 3.77 | <0.001 |
| Middle orbital Frontal |  | R | 33 | 51 | -14 | 164 | 3.72 | <0.001 |
|  | Middle orbital Frontal | R | 38 | 41 | -15 | s.c | 3.66 | <0.001 |
| Middle Cingulum |  | R | 9 | 32 | 31 | 113 | 3.7 | <0.001 |
|  | Anteiror Cingulum | R | 5 | 23 | 27 | s.c | 3.54 | <0.001 |
|  | Superior medial Frontal | R | 6 | 39 | 34 | s.c | 3.35 | 0.001 |
| Postcentral |  | L | -26 | -37 | 58 | 168 | 3.67 | <0.001 |
|  | Precentral | L | -30 | -25 | 58 | s.c | 3.53 | <0.001 |
| Insula |  | L | -35 | 15 | 4 | 132 | 3.59 | <0.001 |
| Superior Occipital |  | R | 23 | -70 | 40 | s.c | 3.85 | <0.001 |
| Middle Temporal |  | L | -56 | -27 | -8 | 106 | 3.85 | <0.001 |
| Middle Frontal |  | R | 29 | 56 | 4 | 106 | 3.77 | <0.001 |
| Middle orbital Frontal |  | R | 33 | 51 | -14 | 164 | 3.72 | <0.001 |
|  | Middle orbital Frontal | R | 38 | 41 | -15 | s.c | 3.66 | <0.001 |
| Middle Cingulum |  | R | 9 | 32 | 31 | 113 | 3.7 | <0.001 |
|  | Anteiror Cingulum | R | 5 | 23 | 27 | s.c | 3.54 | <0.001 |
|  | Superior medial Frontal | R | 6 | 39 | 34 | s.c | 3.35 | 0.001 |
| Postcentral |  | L | -26 | -37 | 58 | 168 | 3.67 | <0.001 |
|  | Precentral | L | -30 | -25 | 58 | s.c | 3.53 | <0.001 |
| Insula |  | L | -35 | 15 | 4 | 132 | 3.59 | <0.001 |

Peak regions are within the Main cluster

Max T is the maximum T statistic for each local maximum. P<0.05 based on non-stationary cluster-extent False discovery rate correction. s.c: same clusters
