# Supplementary material for: Catechol-O-Methyltransferase Val158Met Polymorphism on Striatum Structural Covariance Networks in Alzheimer’s Disease
Source: Mol Neurobiol. 2017 Jul 13;55(6):4637–49. doi: 10.1007/s12035-017-0668-2 (PMC5948254; doi:10.1007/s12035-017-0668-2)
Supplement: Supplementary file 5 — (DOCX 20 kb) [file 12035_2017_668_MOESM4_ESM.docx]

**Supplementary table 3. Structural covariance network for catechol-O-methyltransferase Met carrier with left posterior cingulate cortex as seed**

| **Main Cluster** | **Peak regions** | **Side** | **Stereotaxic coordinates** | | | **Extent** | **Max T** | **P-value** |
| --- | --- | --- | --- | --- | --- | --- | --- | --- |
|  |  | Side | x | y | z |  |  |  |
| Middle Cingulum |  | L | -2 | -36 | 34 | 80635 | 27.72 | <0.001 |
|  | Anteiror Cingulum | L | -3 | 20 | 30 | s.c | 9.94 | <0.001 |
|  | Anteiror Cingulum | R | 5 | 27 | 25 | s.c | 8.87 | <0.001 |
| Inferior Temporal |  | L | -51 | -18 | -35 | 2705 | 5.1 | <0.001 |
|  | Inferior Temporal | L | -35 | -9 | -44 | s.c | 4.54 | <0.001 |
|  | Inferior Temporal | L | -45 | 6 | -39 | s.c | 4.5 | <0.001 |
| undefined |  | R | 0 | -34 | -15 | 110 | 4.15 | <0.001 |
|  | Vermis_3 | R | 5 | -42 | -20 | s.c | 3.07 | 0.001 |
| SupraMarginal |  | L | -60 | -24 | 19 | 124 | 3.61 | <0.001 |
|  | Postcentral | L | -62 | -18 | 28 | s.c | 2.98 | 0.002 |
| Superior Temporal |  | R | 54 | -40 | 18 | 151 | 3.47 | <0.001 |

Peak regions are within the Main cluster

Max T is the maximum T statistic for each local maximum. P<0.05 based on non-stationary cluster-extent False discovery rate correction. s.c: same clusters
