# Supplementary material for: Catechol-O-Methyltransferase Val158Met Polymorphism on Striatum Structural Covariance Networks in Alzheimer’s Disease
Source: Mol Neurobiol. 2017 Jul 13;55(6):4637–49. doi: 10.1007/s12035-017-0668-2 (PMC5948254; doi:10.1007/s12035-017-0668-2)
Supplement: Supplementary file 6 — (DOCX 19 kb) [file 12035_2017_668_MOESM5_ESM.docx]

**Supplementary table 4. Structural covariance network for catechol-O-methyltransferase Valine homozygotes with left posterior cingulate cortex as seed**

| **Main Cluster** | **Peak regions** | **Side** | **Stereotaxic coordinates** | | | **Extent** | **Max T** | **P-value** |
| --- | --- | --- | --- | --- | --- | --- | --- | --- |
|  |  |  | x | y | z |  |  |  |
| Middle Cingulum |  | L | -2 | -36 | 36 | 136933 | 34.56 | <0.001 |
|  | Middle Cingulum | L | -2 | -3 | 39 | s.c | 9.49 | <0.001 |
|  | Angular | L | -50 | -63 | 34 | s.c | 8.83 | <0.001 |
| Hippocampus |  | L | -32 | -30 | -12 | 210 | 3.17 | 0.001 |
|  | ParaHippocampal | L | -30 | -37 | -8 | s.c | 2.98 | 0.002 |

Peak regions are within the Main cluster

Max T is the maximum T statistic for each local maximum. P<0.05 based on non-stationary cluster-extent False discovery rate correction. s.c: same clusters
