# Supplementary material for: Catechol-O-Methyltransferase Val158Met Polymorphism on Striatum Structural Covariance Networks in Alzheimer’s Disease
Source: Mol Neurobiol. 2017 Jul 13;55(6):4637–49. doi: 10.1007/s12035-017-0668-2 (PMC5948254; doi:10.1007/s12035-017-0668-2)
Supplement: Supplementary file 7 — (DOCX 19 kb) [file 12035_2017_668_MOESM6_ESM.docx]

**Supplementary table 5.** **Structural covariance network for catechol-O-methyltransferase Met carrier with right frontoinsular as seed Structural covariance network**

| **Main Cluster** | **Peak regions** | **Side** | **Stereotaxic coordinates** | | | **Extent** | **Max T** | **P-value** |
| --- | --- | --- | --- | --- | --- | --- | --- | --- |
|  |  |  | x | y | z |  |  |  |
| Inferior orbital Frontal |  | R | 36 | 26 | -11 | 121824 | 18.18 | <0.001 |
|  | Insula | R | 41 | 12 | 6 | s.c | 7.45 | <0.001 |
|  | Inferior Temporal | L | -54 | -24 | -29 | s.c | 7.19 | <0.001 |
| Precentral |  | L | -27 | -15 | 64 | 137 | 3.56 | <0.001 |

Peak regions are within the Main cluster

Max T is the maximum T statistic for each local maximum. P<0.05 based on non-stationary cluster-extent False discovery rate correction. s.c: same clusters
