# Supplementary material for: Catechol-O-Methyltransferase Val158Met Polymorphism on Striatum Structural Covariance Networks in Alzheimer’s Disease
Source: Mol Neurobiol. 2017 Jul 13;55(6):4637–49. doi: 10.1007/s12035-017-0668-2 (PMC5948254; doi:10.1007/s12035-017-0668-2)
Supplement: Supplementary file 8 — (DOCX 19 kb) [file 12035_2017_668_MOESM7_ESM.docx]

**Supplementarytable 6. Structural covariance network for catechol-O-methyltransferase Valine homozygotes with right frontoinsular as seed Structural covariance network**

| **Main Cluster** | **Peak regions** | **Side** | **Stereotaxic coordinates** | | | **Extent** | **Max T** | **P-value** |
| --- | --- | --- | --- | --- | --- | --- | --- | --- |
|  |  |  | x | y | z |  |  |  |
| Inferior orbital Frontal |  | R | 36 | 26 | -11 | 155825 | 17.23 | <0.001 |
|  | Superior Frontal | R | 17 | 63 | 10 | s.c | 7.34 | <0.001 |
|  | undefined | R | 36 | 12 | 0 | s.c | 7.28 | <0.001 |
| Middle Occipital |  | R | 30 | -85 | 9 | 136 | 3.05 | 0.001 |

Peak regions are within the Main cluster

Max T is the maximum T statistic for each local maximum. P<0.05 based on non-stationary cluster-extent False discovery rate correction.s.c: same clusters
