# Supplementary material for: Catechol-O-Methyltransferase Val158Met Polymorphism on Striatum Structural Covariance Networks in Alzheimer’s Disease
Source: Mol Neurobiol. 2017 Jul 13;55(6):4637–49. doi: 10.1007/s12035-017-0668-2 (PMC5948254; doi:10.1007/s12035-017-0668-2)
Supplement: Supplementary file 9 — (DOCX 18 kb) [file 12035_2017_668_MOESM8_ESM.docx]

**Supplementary table 7.** **Structural covariance network for catechol-O-methyltransferase Met carrier with left dorsolateral prefrontal cortex as seed**

| **Main Cluster** | **Peak regions** | **Side** | **Stereotaxic coordinates** | | | **Extent** | **Max T** | **P-value** |
| --- | --- | --- | --- | --- | --- | --- | --- | --- |
|  |  |  | x | y | z |  |  |  |
| Middle Frontal |  | R | 42 | 36 | 19 | 1267 | 16.28 | <0.001 |
|  | Middle Frontal | R | 44 | 47 | 1 | s.c | 4.31 | <0.001 |

Peak regions are within the Main cluster

Max T is the maximum T statistic for each local maximum. P<0.05 based on non-stationary cluster-extent False discovery rate correction.s.c: same clusters
