# Supplementary material for: Catechol-O-Methyltransferase Val158Met Polymorphism on Striatum Structural Covariance Networks in Alzheimer’s Disease
Source: Mol Neurobiol. 2017 Jul 13;55(6):4637–49. doi: 10.1007/s12035-017-0668-2 (PMC5948254; doi:10.1007/s12035-017-0668-2)
Supplement: Supplementary file 10 — (DOCX 21 kb) [file 12035_2017_668_MOESM9_ESM.docx]

**Supplementary table 8. Structural covariance network for catechol-O-methyltransferase Valine homozygotes with left dorsolateral prefrontal cortex as seed**

| **Main Cluster** | **Peak regions** | **Side** | **Stereotaxic coordinates** | | | | | **Extent** | **Max T** | **P-value** |
| --- | --- | --- | --- | --- | --- | --- | --- | --- | --- | --- |
|  |  |  | x | | y | | z |  |  |  |
| Middle Frontal |  | R | 42 | 36 | | 19 | | 23050 | 16.74 | <0.001 |
|  | Middle Frontal | R | 42 | 44 | | 6 | | s.c | 7.4 | <0.001 |
|  | Precentral | R | 45 | 3 | | 42 | | s.c | 6.8 | <0.001 |
| Frontal inferior operculum |  | L | -45 | 5 | | 27 | | 27649 | 7.66 | <0.001 |
|  | Frontal inferio triangular region | L | -41 | 32 | | 24 | | s.c | 6.64 | <0.001 |
|  | Frontal inferio triangular region | L | -41 | 41 | | 13 | | s.c | 6.3 | <0.001 |
| Inferior Temporal |  | L | -50 | -19 | | -29 | | 292 | 5.3 | <0.001 |
| Precuneus |  | R | 17 | -64 | | 46 | | 236 | 4.84 | <0.001 |
|  | Superior Occipital | R | 27 | -61 | | 37 | | s.c | 3.16 | 0.001 |
| Middle Cingulum |  | R | 3 | -28 | | 39 | | 3310 | 4.54 | <0.001 |
|  | Middle Cingulum | R | 8 | 0 | | 37 | | s.c | 4.34 | <0.001 |
|  | Middle Cingulum | R | 11 | -25 | | 45 | | s.c | 4.27 | <0.001 |
| Inferior Temporal |  | R | 50 | -11 | | -33 | | 297 | 4.31 | <0.001 |
|  | Inferior Temporal | R | 48 | -2 | | -39 | | s.c | 3.22 | 0.001 |
| Fusiform |  | L | -44 | -40 | | -20 | | 169 | 4.24 | <0.001 |
| Middle Temporal |  | R | 54 | -10 | | -17 | | 389 | 4.2 | <0.001 |
|  | Middle Temporal | R | 53 | -21 | | -11 | | s.c | 3.3 | 0.001 |
| Caudate |  | L | -9 | 5 | | 13 | | 292 | 4.11 | <0.001 |
|  | Caudate | L | -6 | 9 | | 4 | | s.c | 3.72 | <0.001 |
| Vermis_3 |  | L | -2 | -37 | | -12 | | 168 | 4.01 | <0.001 |
| Middle Temporal |  | R | 47 | -61 | | 16 | | 300 | 4 | <0.001 |
| undefined |  | L | -24 | -42 | | 0 | | 155 | 3.58 | <0.001 |
|  | Hippocampus | L | -30 | -37 | | -5 | | s.c | 3.56 | <0.001 |
|  | Hippocampus | L | -35 | -30 | | -11 | | s.c | 3.48 | <0.001 |

Peak regions are within the Main cluster

Max T is the maximum T statistic for each local maximum. P<0.05 based on non-stationary cluster-extent False discovery rate correction.s.c: same clusters
