# Supplementary material for: Catechol-O-Methyltransferase Val158Met Polymorphism on Striatum Structural Covariance Networks in Alzheimer’s Disease
Source: Mol Neurobiol. 2017 Jul 13;55(6):4637–49. doi: 10.1007/s12035-017-0668-2 (PMC5948254; doi:10.1007/s12035-017-0668-2)
Supplement: Supplementary file 11 — (DOCX 21 kb) [file 12035_2017_668_MOESM10_ESM.docx]

**Supplementary table 9. Structural covariance network for catechol-O-methyltransferase Met carriers with right ventral inferior caudate as seed**

| **Main Cluster** | **Peak regions** | **Side** | **Stereotaxic coordinates** | | | **Extent** | **Max T** | **P-value** |
| --- | --- | --- | --- | --- | --- | --- | --- | --- |
|  |  |  | x | y | z |  |  |  |
| Olfactory |  | R | 9 | 9 | -18 | 39711 | 32.13 | <0.001 |
|  | Olfactory | L | -8 | 8 | -17 | s.c | 8.2 | <0.001 |
|  | Frontal inferior operculum | R | 57 | 12 | 25 | s.c | 6.91 | <0.001 |
| Inferior Temporal |  | L | -45 | -22 | -32 | 603 | 6.34 | <0.001 |
|  | Inferior Temporal | L | -54 | -40 | -29 | s.c | 5.06 | <0.001 |
|  | Inferior Temporal | L | -60 | -46 | -24 | s.c | 4.69 | <0.001 |
| Lingual |  | L | -6 | -61 | 1 | 229 | 5.6 | <0.001 |
| Frontal inferior operculum |  | L | -53 | 9 | 25 | 200 | 5.49 | <0.001 |
|  | Precentral | L | -56 | -1 | 36 | s.c | 3.99 | <0.001 |
| Superior Parietal |  | L | -23 | -72 | 46 | 769 | 5.48 | <0.001 |
|  | Middle Occipital | L | -26 | -79 | 40 | s.c | 5.43 | <0.001 |
|  | Superior Occipital | L | -11 | -79 | 43 | s.c | 5.38 | <0.001 |
| Superior Frontal |  | R | 23 | 6 | 66 | 106 | 4.96 | <0.001 |
|  | Superior Frontal | R | 21 | -12 | 70 | s.c | 4.26 | <0.001 |
| Precuneus |  | R | 17 | -78 | 46 | 198 | 4.87 | <0.001 |
|  | Superior Parietal | R | 24 | -73 | 48 | s.c | 4.37 | <0.001 |
| Middle Temporal |  | L | -57 | -63 | 7 | 158 | 4.73 | <0.001 |
|  | Middle Temporal | L | -56 | -64 | -2 | s.c | 4.2 | <0.001 |
|  | Inferior Temporal | L | -53 | -69 | -8 | s.c | 4.18 | <0.001 |
| Fusiform |  | L | -24 | -40 | -17 | 183 | 4.57 | <0.001 |
|  | Lingual | L | -17 | -54 | -11 | s.c | 4.18 | <0.001 |
| SupraMarginal gyrus |  | R | 62 | -21 | 18 | 132 | 4.46 | <0.001 |
| Thalamus |  | L | -14 | -31 | 1 | 174 | 4.43 | <0.001 |

Peak regions are within the Main cluster

Max T is the maximum T statistic for each local maximum. FDR P<0.0001 based on non-stationary cluster-extent False discovery rate correction. s.c: same clusters
