# Supplementary material for: Catechol-O-Methyltransferase Val158Met Polymorphism on Striatum Structural Covariance Networks in Alzheimer’s Disease
Source: Mol Neurobiol. 2017 Jul 13;55(6):4637–49. doi: 10.1007/s12035-017-0668-2 (PMC5948254; doi:10.1007/s12035-017-0668-2)
Supplement: Supplementary file 12 — (DOCX 20 kb) [file 12035_2017_668_MOESM11_ESM.docx]

**Supplementary table 10. Structural covariance network for catechol-O-methyltransferase Valine homozygotes with right ventral inferior caudate as seed**

| **Main Cluster** | **Peak regions** | **Side** | **Stereotaxic coordinates** | | | **Extent** | **Max T** | **P-value** |
| --- | --- | --- | --- | --- | --- | --- | --- | --- |
|  |  |  | x | y | z |  |  |  |
| Olfactory |  | R | 9 | 9 | -18 | 79118 | 32.3 | <0.001 |
|  | undefined | L | -8 | 8 | -18 | s.c | 10.34 | <0.001 |
|  | Rectus | L | -2 | 18 | -24 | s.c | 9.13 | <0.001 |
| Superior Frontal |  | R | 26 | 2 | 60 | 164 | 4.77 | <0.001 |
|  | Superior Frontal | R | 20 | 14 | 63 | s.c | 4.35 | <0.001 |
| Lingual |  | R | 9 | -52 | 6 | 132 | 4.44 | <0.001 |
| Lingual |  | L | -11 | -61 | 1 | 208 | 4.37 | <0.001 |
| Superior Parietal |  | L | -14 | -76 | 49 | 246 | 4.32 | <0.001 |
|  | Precuneus | L | -14 | -72 | 58 | s.c | 4.32 | <0.001 |
|  | Precuneus | L | -11 | -60 | 64 | s.c | 4.14 | <0.001 |

Peak regions are within the Main cluster

Max T is the maximum T statistic for each local maximum. FDR P<0.0001 based on non-stationary cluster-extent False discovery rate correction. s.c: same clusters
