# Supplementary material for: Catechol-O-Methyltransferase Val158Met Polymorphism on Striatum Structural Covariance Networks in Alzheimer’s Disease
Source: Mol Neurobiol. 2017 Jul 13;55(6):4637–49. doi: 10.1007/s12035-017-0668-2 (PMC5948254; doi:10.1007/s12035-017-0668-2)
Supplement: Supplementary file 13 — (DOCX 19 kb) [file 12035_2017_668_MOESM12_ESM.docx]

**Supplementary table 11. Structural covariance network for catechol-O-methyltransferase Met carriers with right ventral superior caudate as seed**

| **Main Cluster** | **Peak regions** | **Side** | **Stereotaxic coordinates** | | | **Extent** | **Max T** | **P-value** |
| --- | --- | --- | --- | --- | --- | --- | --- | --- |
|  |  |  | x | y | z |  |  |  |
| Caudate |  | R | 11 | 15 | 0 | 124015 | 56.81 | <0.001 |
|  | Caudate | L | -8 | 15 | 0 | s.c | 14.47 | <0.001 |

Peak regions are within the Main cluster

Max T is the maximum T statistic for each local maximum. FDR P<0.0001 based on non-stationary cluster-extent False discovery rate correction. s.c: same clusters
