# Supplementary material for: Catechol-O-Methyltransferase Val158Met Polymorphism on Striatum Structural Covariance Networks in Alzheimer’s Disease
Source: Mol Neurobiol. 2017 Jul 13;55(6):4637–49. doi: 10.1007/s12035-017-0668-2 (PMC5948254; doi:10.1007/s12035-017-0668-2)
Supplement: Supplementary file 14 — (DOCX 20 kb) [file 12035_2017_668_MOESM13_ESM.docx]

**Supplementary table 12. Structural covariance network for catechol-O-methyltransferase Valine homozygotes with right ventral superior caudate as seed**

| **Main Cluster** | **Peak regions** | **Side** | **Stereotaxic coordinates** | | | **Extent** | **Max T** | **P-value** |
| --- | --- | --- | --- | --- | --- | --- | --- | --- |
|  |  |  | x | y | z |  |  |  |
| Caudate |  | R | 11 | 14 | 0 | 33.82 | 81262 | <0.001 |
|  | Caudate | L | -9 | 15 | -5 | 12.67 | s.c | <0.001 |
|  | Caudate | R | 11 | 2 | 15 | 10.97 | s.c | <0.001 |
| Superior Occipital |  | L | -18 | -85 | 30 | 6.32 | 396 | <0.001 |
|  | Superior Occipital | L | -14 | -94 | 18 | 4.22 | s.c | <0.001 |
| Cerebelum_6_R |  | R | 23 | -66 | -17 | 6.27 | 1096 | <0.001 |
|  | Fusiform | R | 24 | -51 | -14 | 5.55 | s.c | <0.001 |
| Precuneus |  | R | 8 | -60 | 63 | 4.69 | 134 | <0.001 |
|  | Superior Parietal | R | 14 | -54 | 71 | 4.66 | s.c | <0.001 |

Peak regions are within the Main cluster

Max T is the maximum T statistic for each local maximum. FDR P<0.0001 based on non-stationary cluster-extent False discovery rate correction. s.c: same clusters
