# Supplementary material for: Catechol-O-Methyltransferase Val158Met Polymorphism on Striatum Structural Covariance Networks in Alzheimer’s Disease
Source: Mol Neurobiol. 2017 Jul 13;55(6):4637–49. doi: 10.1007/s12035-017-0668-2 (PMC5948254; doi:10.1007/s12035-017-0668-2)
Supplement: Supplementary file 15 — (DOCX 20 kb) [file 12035_2017_668_MOESM14_ESM.docx]

**Supplementary table 13. Structural covariance network for catechol-O-methyltransferase Met carriers with right dorsal caudate nucleus as seed**

| **Main Cluster** | **Peak regions** | **Side** | **Stereotaxic coordinates** | | | **Extent** | **Max T** | **P-value** |
| --- | --- | --- | --- | --- | --- | --- | --- | --- |
|  |  |  | x | y | z |  |  |  |
| Caudate |  | R | 14 | 15 | 9 | 64028 | 62.39 | <0.001 |
|  | Caudate | L | -12 | 14 | 7 | s.c | 14.85 | <0.001 |
|  | Caudate | L | -6 | 6 | 6 | s.c | 12.43 | <0.001 |
| SupraMarginal gyrus |  | R | 60 | -22 | 33 | 1241 | 5.26 | <0.001 |
|  | Precentral | R | 54 | -15 | 43 | s.c | 5.07 | <0.001 |
|  | Precentral | R | 38 | -16 | 46 | s.c | 4.3 | <0.001 |
| Inferior Temporal |  | R | 56 | -35 | -26 | 929 | 5.06 | <0.001 |
|  | Inferior Temporal | R | 50 | -21 | -29 | s.c | 5.05 | <0.001 |
| Frontal inferior operculum |  | L | -51 | 14 | 21 | 259 | 4.95 | <0.001 |
| Inferior Temporal |  | L | -50 | -27 | -29 | 307 | 4.76 | <0.001 |
|  | Inferior Temporal | L | -44 | -45 | -24 | s.c | 4.34 | <0.001 |
| Hippocampus |  | L | -29 | -37 | -3 | 146 | 4.5 | <0.001 |
|  | Hippocampus | L | -26 | -37 | 6 | s.c | 4.23 | <0.001 |
| Middle Temporal |  | R | 47 | -72 | 1 | 314 | 4.19 | <0.001 |
|  | Middle Temporal | R | 54 | -67 | 13 | s.c | 4.16 | <0.001 |
| Hippocampus |  | R | 14 | -34 | 9 | 202 | 4.17 | <0.001 |
|  | Lingual | R | 15 | -31 | -6 | s.c | 4.07 | <0.001 |

Peak regions are within the Main cluster

Max T is the maximum T statistic for each local maximum. FDR P<0.0001 based on non-stationary cluster-extent False discovery rate correction. s.c: same clusters
