# Supplementary material for: Catechol-O-Methyltransferase Val158Met Polymorphism on Striatum Structural Covariance Networks in Alzheimer’s Disease
Source: Mol Neurobiol. 2017 Jul 13;55(6):4637–49. doi: 10.1007/s12035-017-0668-2 (PMC5948254; doi:10.1007/s12035-017-0668-2)
Supplement: Supplementary file 16 — (DOCX 21 kb) [file 12035_2017_668_MOESM15_ESM.docx]

**Supplementary table 14. Structural covariance network for catechol-O-methyltransferase Valine homozygotes with right dorsal caudate nucleus as seed**

| **Main Cluster** | **Peak regions** | **Side** | **Stereotaxic coordinates** | | | **Extent** | **Max T** | **P-value** |
| --- | --- | --- | --- | --- | --- | --- | --- | --- |
|  |  |  | x | y | z |  |  |  |
| Caudate |  | R | 12 | 15 | 7 | 44974 | 30.19 | <0.001 |
|  | Caudate | L | -11 | 11 | 9 | s.c | 10.38 | <0.001 |
|  | Caudate | L | -11 | 18 | -6 | s.c | 9.71 | <0.001 |
| Inferior Temporal |  | L | -48 | -28 | -27 | 767 | 5.57 | <0.001 |
|  | Fusiform | L | -39 | -51 | -20 | s.c | 4.41 | <0.001 |
| Superior Frontal |  | L | -21 | 17 | 52 | 397 | 5.25 | <0.001 |
|  | Superior Frontal | L | -18 | 21 | 58 | s.c | 5.11 | <0.001 |
|  | Superior Frontal | L | -20 | 12 | 60 | s.c | 4.48 | <0.001 |
| Inferior Temporal |  | R | 48 | -24 | -29 | 282 | 5.25 | <0.001 |
|  | Inferior Temporal | R | 57 | -29 | -29 | s.c | 4.14 | <0.001 |
| Middle Cingulum |  | L | -11 | -30 | 45 | 558 | 5.21 | <0.001 |
| Lingual |  | L | -9 | -76 | 0 | 1393 | 5.04 | <0.001 |
|  | Lingual | L | -11 | -63 | 0 | s.c | 4.86 | <0.001 |
|  | Calcarine | L | -8 | -61 | 10 | s.c | 4.63 | <0.001 |
| Middle Temporal |  | L | -54 | -4 | -18 | 322 | 4.64 | <0.001 |
|  | Superior Temporal Pole | L | -54 | 6 | -14 | s.c | 4.03 | <0.001 |
| Middle Frontal |  | R | 27 | 9 | 57 | 213 | 4.35 | <0.001 |
|  | Superior Frontal | R | 26 | -7 | 63 | s.c | 4.19 | <0.001 |
|  | Middle Frontal | R | 27 | 20 | 54 | s.c | 4.01 | <0.001 |
| Middle Temporal |  | R | 53 | -69 | 19 | 756 | 4.35 | <0.001 |
|  | Inferior Temporal | R | 54 | -69 | -3 | s.c | 4.35 | <0.001 |
|  | Middle Temporal | R | 48 | -64 | 10 | s.c | 4.28 | <0.001 |
| Lingual |  | R | 12 | -66 | 4 | 271 | 4.28 | <0.001 |
|  | Lingual | R | 9 | -55 | 7 | s.c | 4.17 | <0.001 |
| Superior Frontal Medial |  | L | -11 | 48 | 4 | 142 | 4.26 | <0.001 |

Peak regions are within the Main cluster

Max T is the maximum T statistic for each local maximum. FDR P<0.0001 based on non-stationary cluster-extent False discovery rate correction. s.c: same clusters
