# Supplementary material for: Catechol-O-Methyltransferase Val158Met Polymorphism on Striatum Structural Covariance Networks in Alzheimer’s Disease
Source: Mol Neurobiol. 2017 Jul 13;55(6):4637–49. doi: 10.1007/s12035-017-0668-2 (PMC5948254; doi:10.1007/s12035-017-0668-2)
Supplement: Supplementary file 17 — (DOCX 21 kb) [file 12035_2017_668_MOESM16_ESM.docx]

**Supplementary table 15. Structural covariance network for catechol-O-methyltransferase Met carriers with right dorsal caudal putamen as seed**

| **Main Cluster** | **Peak regions** | **Side** | **Stereotaxic coordinates** | | | **Extent** | **Max T** | **P-value** |
| --- | --- | --- | --- | --- | --- | --- | --- | --- |
|  |  |  | x | y | z |  |  |  |
| Putamen |  | R | 30 | 2 | 3 | 29075 | 39.54 | <0.001 |
|  | Putamen | R | 30 | -3 | -5 | s.c | 19.75 | <0.001 |
|  | undefined | R | 24 | 3 | -11 | s.c | 16.42 | <0.001 |
| Paracentral Lobule |  | L | -15 | -25 | 66 | 10601 | 8.26 | <0.001 |
|  | undefined | L | -17 | -34 | 58 | s.c | 7.91 | <0.001 |
|  | Postcentral | R | 15 | -34 | 63 | s.c | 7.55 | <0.001 |
| undefined |  | L | -47 | -27 | -30 | 390 | 8.01 | <0.001 |
|  | Inferior Temporal | L | -47 | -19 | -35 | s.c | 4.99 | <0.001 |
| undefined |  | L | -24 | -33 | 6 | 959 | 6.05 | <0.001 |
|  | Lingual | L | -14 | -39 | -8 | s.c | 6 | <0.001 |
|  | undefined | L | -18 | -30 | -5 | s.c | 5.64 | <0.001 |
| Inferior Parietal |  | R | 48 | -57 | 49 | 847 | 5.33 | <0.001 |
|  | Inferior Parietal | R | 59 | -40 | 46 | s.c | 4.94 | <0.001 |
|  | Inferior Parietal | R | 56 | -54 | 43 | s.c | 4.83 | <0.001 |
| Inferior Parietal |  | L | -36 | -52 | 48 | 313 | 5.03 | <0.001 |
|  | Inferior Parietal | L | -44 | -37 | 46 | s.c | 4.66 | <0.001 |
|  | undefined | L | -23 | -55 | 42 | s.c | 4.45 | <0.001 |
| Superior Occipital |  | L | -15 | -84 | 28 | 1024 | 4.99 | <0.001 |
|  | Superior Parietal | L | -21 | -72 | 51 | s.c | 4.93 | <0.001 |
|  | Lingual | L | -17 | -81 | -3 | s.c | 4.84 | <0.001 |
| undefined |  | R | 41 | -58 | 6 | 133 | 4.96 | <0.001 |
|  | Middle Temporal | R | 45 | -54 | -2 | s.c | 4.09 | <0.001 |
|  | Inferior Temporal | R | 50 | -52 | -9 | s.c | 3.99 | <0.001 |
| Superior Temporal |  | L | -62 | -42 | 22 | 119 | 4.64 | <0.001 |
|  | Superior Temporal | L | -60 | -36 | 15 | s.c | 3.87 | <0.001 |
|  | SupraMarginal gyrus | L | -60 | -39 | 36 | s.c | 3.84 | <0.001 |
| Precentral |  | R | 56 | -10 | 45 | 106 | 4.36 | <0.001 |
|  | Postcentral | R | 59 | -18 | 43 | s.c | 4.32 | <0.001 |

Peak regions are within the Main cluster

Max T is the maximum T statistic for each local maximum. FDR P<0.0001 based on non-stationary cluster-extent False discovery rate correction. s.c: same clusters
