# Supplementary material for: Catechol-O-Methyltransferase Val158Met Polymorphism on Striatum Structural Covariance Networks in Alzheimer’s Disease
Source: Mol Neurobiol. 2017 Jul 13;55(6):4637–49. doi: 10.1007/s12035-017-0668-2 (PMC5948254; doi:10.1007/s12035-017-0668-2)
Supplement: Supplementary file 18 — (DOCX 22 kb) [file 12035_2017_668_MOESM17_ESM.docx]

**Supplementary table 16. Structural covariance network for catechol-O-methyltransferase Valine homozygotes with right dorsal caudal putamen as seed**

| **Main Cluster** | **Peak regions** | **Side** | **Stereotaxic coordinates** | | | **Extent** | **Max T** | **P-value** |
| --- | --- | --- | --- | --- | --- | --- | --- | --- |
|  |  |  | x | y | z |  |  |  |
| Putamen |  | R | 30 | 2 | 3 | 19642 | 31.01 | <0.001 |
|  | Putamen | R | 27 | 0 | -8 | s.c | 15.01 | <0.001 |
|  | Putamen | L | -27 | 5 | 6 | s.c | 13.25 | <0.001 |
| Inferior Temporal |  | R | 48 | -27 | -29 | 608 | 8.08 | <0.001 |
| Inferior Temporal |  | L | -45 | -30 | -29 | 287 | 5.94 | <0.001 |
|  | undefined | L | -45 | -22 | -33 | s.c | 5.62 | <0.001 |
|  | Fusiform | L | -32 | -27 | -29 | s.c | 3.99 | <0.001 |
| Superior Frontal Medial |  | R | 6 | 51 | 4 | 225 | 5.67 | <0.001 |
|  | Anteiror Cingulum | R | 6 | 44 | 13 | s.c | 4.34 | <0.001 |
|  | Superior Frontal Medial | R | 6 | 60 | 3 | s.c | 4.25 | <0.001 |
| Lingual |  | L | -12 | -75 | 0 | 168 | 5.48 | <0.001 |
|  | Lingual | L | -12 | -85 | -3 | s.c | 4.65 | <0.001 |
| SupraMarginal gyrus |  | R | 60 | -45 | 42 | 198 | 5.25 | <0.001 |
|  | SupraMarginal gyrus | R | 65 | -37 | 39 | s.c | 4.92 | <0.001 |
| Superior Occipital |  | L | -14 | -85 | 27 | 126 | 5.23 | <0.001 |
|  | Calcarine | L | -11 | -88 | 12 | s.c | 4.75 | <0.001 |
| Thalamus |  | L | -8 | -19 | -2 | 186 | 5.18 | <0.001 |
|  | Thalamus | L | -18 | -27 | 7 | s.c | 4.87 | <0.001 |
|  | undefined | L | -15 | -28 | -3 | s.c | 4.42 | <0.001 |
| Middle Frontal |  | L | -33 | 42 | 33 | 362 | 5.11 | <0.001 |
|  | Superior orbital frontal | L | -27 | 57 | -5 | s.c | 5.04 | <0.001 |
|  | Middle Frontal | L | -38 | 30 | 42 | s.c | 4.94 | <0.001 |
| Calcarine |  | R | 21 | -88 | 0 | 131 | 5.04 | <0.001 |
|  | Calcarine | R | 17 | -85 | 12 | s.c | 4.56 | <0.001 |
| Postcentral |  | L | -53 | -10 | 15 | 676 | 4.98 | <0.001 |
|  | Superior Temporal | L | -54 | -24 | 9 | s.c | 4.82 | <0.001 |
|  | Heschl | L | -62 | -12 | 7 | s.c | 4.72 | <0.001 |
| Superior Temporal |  | R | 60 | -57 | 19 | 350 | 4.91 | <0.001 |
|  | Middle Temporal | R | 56 | -63 | 10 | s.c | 4.24 | <0.001 |
|  | Middle Temporal | R | 51 | -73 | 15 | s.c | 4.18 | <0.001 |
| Inferior Temporal |  | R | 54 | -69 | -3 | 121 | 4.8 | <0.001 |

Peak regions are within the Main cluster

Max T is the maximum T statistic for each local maximum. FDR P<0.0001 based on non-stationary cluster-extent False discovery rate correction. s.c: same clusters
