# Supplementary material for: Catechol-O-Methyltransferase Val158Met Polymorphism on Striatum Structural Covariance Networks in Alzheimer’s Disease
Source: Mol Neurobiol. 2017 Jul 13;55(6):4637–49. doi: 10.1007/s12035-017-0668-2 (PMC5948254; doi:10.1007/s12035-017-0668-2)
Supplement: Supplementary file 19 — (DOCX 20 kb) [file 12035_2017_668_MOESM18_ESM.docx]

**Supplementary table 17. Structural covariance network for catechol-O-methyltransferase Met carriers with right dorsal rostral putamen as seed**

| **Main Cluster** | **Peak regions** | **Side** | **Stereotaxic coordinates** | | | **Extent** | **Max T** | **P-value** |
| --- | --- | --- | --- | --- | --- | --- | --- | --- |
|  |  |  | x | y | z |  |  |  |
| Putamen |  | R | 29 | 9 | 6 | 57672 | 28.71 | <0.001 |
|  | Putamen | R | 23 | 17 | 3 | s.c | 17.81 | <0.001 |
|  | undefined | R | 26 | 2 | -9 | s.c | 16.23 | <0.001 |
| Inferior Temporal |  | L | -48 | -28 | -29 | 552 | 7.64 | <0.001 |
|  | Inferior Temporal | L | -42 | -39 | -27 | s.c | 5.17 | <0.001 |
| undefined |  | L | -26 | -34 | 4 | 1119 | 5.85 | <0.001 |
|  | Lingual | L | -14 | -37 | -8 | s.c | 5.81 | <0.001 |
|  | Lingual | L | -8 | -55 | 1 | s.c | 5.3 | <0.001 |
| Frontal inferior operculum |  | L | -51 | 12 | 25 | 123 | 4.59 | <0.001 |
|  | Frontal inferior operculum | L | -56 | 12 | 16 | s.c | 4.38 | <0.001 |

Peak regions are within the Main cluster

Max T is the maximum T statistic for each local maximum. FDR P<0.0001 based on non-stationary cluster-extent False discovery rate correction. s.c: same clusters
