# Supplementary material for: Catechol-O-Methyltransferase Val158Met Polymorphism on Striatum Structural Covariance Networks in Alzheimer’s Disease
Source: Mol Neurobiol. 2017 Jul 13;55(6):4637–49. doi: 10.1007/s12035-017-0668-2 (PMC5948254; doi:10.1007/s12035-017-0668-2)
Supplement: Supplementary file 20 — (DOCX 22 kb) [file 12035_2017_668_MOESM19_ESM.docx]

**Supplementary table 18. Structural covariance network for catechol-O-methyltransferase Valine homozygotes with right dorsal rostral putamen as seed**

| **Main Cluster** | **Peak regions** | **Side** | **Stereotaxic coordinates** | | | **Extent** | **Max T** | **P-value** |
| --- | --- | --- | --- | --- | --- | --- | --- | --- |
|  |  |  | x | y | z |  |  |  |
| Putamen |  | R | 29 | 8 | 6 | 25658 | 32.7 | <0.001 |
|  | Putamen | L | -27 | 5 | 6 | s.c | 15.97 | <0.001 |
|  | undefined | R | 26 | 17 | -9 | s.c | 15.74 | <0.001 |
| Inferior Temporal |  | R | 48 | -27 | -29 | 533 | 8.37 | <0.001 |
| undefined |  | L | -45 | -27 | -30 | 455 | 6.91 | <0.001 |
|  | Inferior Temporal | L | -50 | -30 | -18 | s.c | 5.18 | <0.001 |
| SupraMarginal gyrus |  | R | 59 | -46 | 42 | 2079 | 6.54 | <0.001 |
|  | Postcentral | R | 57 | -18 | 40 | s.c | 6.01 | <0.001 |
|  | Angular | R | 59 | -60 | 24 | s.c | 5.56 | <0.001 |
| Superior Frontal Medial |  | L | -14 | 48 | 7 | 303 | 6.36 | <0.001 |
| undefined |  | R | 3 | -39 | -24 | 256 | 6.33 | <0.001 |
| Middle Frontal |  | L | -39 | 44 | 25 | 1300 | 6.2 | <0.001 |
|  | Middle Frontal | L | -30 | 50 | 24 | s.c | 5.89 | <0.001 |
|  | Middle Frontal | L | -38 | 30 | 42 | s.c | 5.77 | <0.001 |
| Superior Occipital |  | L | -14 | -84 | 25 | 236 | 6.04 | <0.001 |
| Lingual |  | L | -12 | -75 | 0 | 515 | 6 | <0.001 |
|  | Lingual | L | -17 | -67 | -2 | s.c | 5.58 | <0.001 |
|  | Lingual | L | -8 | -58 | 0 | s.c | 5.18 | <0.001 |
| Superior Frontal Medial |  | R | 6 | 50 | 6 | 301 | 5.97 | <0.001 |
|  | Superior Frontal Medial | R | 6 | 60 | 3 | s.c | 4.87 | <0.001 |
|  | Anteiror Cingulum | R | 2 | 44 | 9 | s.c | 4.17 | <0.001 |
| Precentral |  | L | -51 | 9 | 30 | 116 | 5.29 | <0.001 |
|  | Frontal inferior operculum | L | -56 | 11 | 19 | s.c | 4.38 | <0.001 |
| Thalamus |  | L | -8 | -18 | 0 | 321 | 5.09 | <0.001 |
|  | undefined | L | 0 | -25 | 0 | s.c | 4.72 | <0.001 |
|  | Thalamus | L | -18 | -27 | 9 | s.c | 4.56 | <0.001 |
| Superior Frontal |  | R | 23 | 54 | 30 | 255 | 4.93 | <0.001 |
|  | Superior Frontal | R | 20 | 63 | 15 | s.c | 4.76 | <0.001 |
|  | Superior Frontal | R | 18 | 60 | 22 | s.c | 4.65 | <0.001 |
| Supplementary Motor Area |  | R | 11 | -19 | 60 | 148 | 4.9 | <0.001 |
|  | Paracentral Lobule | R | 12 | -30 | 57 | s.c | 4.66 | <0.001 |
|  | Supplementary Motor Area | R | 12 | -22 | 67 | s.c | 4.34 | <0.001 |
| Lingual |  | R | 15 | -82 | -2 | 199 | 4.82 | <0.001 |
|  | Lingual | R | 15 | -70 | 1 | s.c | 4.7 | <0.001 |
|  | Fusiform | R | 27 | -82 | -3 | s.c | 4.48 | <0.001 |
| Fusiform |  | L | -24 | -72 | -17 | 150 | 4.45 | <0.001 |
|  | Fusiform | L | -20 | -42 | -14 | s.c | 4.28 | <0.001 |

Peak regions are within the Main cluster

Max T is the maximum T statistic for each local maximum. FDR P<0.0001 based on non-stationary cluster-extent False discovery rate correction. s.c: same clusters
