# Supplementary material for: Catechol-O-Methyltransferase Val158Met Polymorphism on Striatum Structural Covariance Networks in Alzheimer’s Disease
Source: Mol Neurobiol. 2017 Jul 13;55(6):4637–49. doi: 10.1007/s12035-017-0668-2 (PMC5948254; doi:10.1007/s12035-017-0668-2)
Supplement: Supplementary file 21 — (DOCX 20 kb) [file 12035_2017_668_MOESM20_ESM.docx]

**Supplementary table 19. Structural covariance network for catechol-O-methyltransferase Met carriers with right ventral rostral putamen as seed**

| **Main Cluster** | **Peak regions** | **Side** | **Stereotaxic coordinates** | | | **Extent** | **Max T** | **P-value** |
| --- | --- | --- | --- | --- | --- | --- | --- | --- |
|  |  |  | x | y | z |  |  |  |
| Putamen |  | R | 21 | 17 | 0 | 80828 | 32.19 | <0.001 |
|  | Putamen | R | 24 | 14 | -11 | s.c | 27.81 | <0.001 |
|  | Putamen | R | 29 | 11 | 3 | s.c | 22.48 | <0.001 |
| Superior Frontal |  | R | 17 | 50 | 39 | 412 | 5.32 | <0.001 |
|  | Superior Frontal | R | 26 | 63 | 3 | s.c | 4.65 | <0.001 |
|  | Superior Frontal | R | 18 | 57 | 27 | s.c | 4.13 | <0.001 |
| Hippocampus |  | L | -29 | -34 | 0 | 529 | 5.21 | <0.001 |
|  | undefined | L | -24 | -34 | 7 | s.c | 4.79 | <0.001 |
|  | Hippocampus | L | -18 | -30 | -6 | s.c | 4.6 | <0.001 |
| Frontal inferior operculum |  | R | 54 | 12 | 22 | 395 | 4.55 | <0.001 |
|  | Frontal inferio triangular region | R | 51 | 26 | 22 | s.c | 4.25 | <0.001 |
|  | Frontal inferior operculum | R | 48 | 15 | 33 | s.c | 4.17 | <0.001 |
| Anteiror Cingulum |  | L | -2 | 35 | 27 | 204 | 4.42 | <0.001 |
|  | Middle Cingulum | L | 0 | 27 | 34 | s.c | 4.3 | <0.001 |

Peak regions are within the Main cluster

Max T is the maximum T statistic for each local maximum. FDR P<0.0001 based on non-stationary cluster-extent False discovery rate correction. s.c: same clusters
