# Supplementary material for: Catechol-O-Methyltransferase Val158Met Polymorphism on Striatum Structural Covariance Networks in Alzheimer’s Disease
Source: Mol Neurobiol. 2017 Jul 13;55(6):4637–49. doi: 10.1007/s12035-017-0668-2 (PMC5948254; doi:10.1007/s12035-017-0668-2)
Supplement: Supplementary file 22 — (DOCX 21 kb) [file 12035_2017_668_MOESM21_ESM.docx]

**Supplementary table 20. Structural covariance network for catechol-O-methyltransferase Valine homozygotes with right ventral rostral putamen as seed**

| **Main Cluster** | **Peak regions** | **Side** | **Stereotaxic coordinates** | | | **Extent** | **Max T** | **P-value** |
| --- | --- | --- | --- | --- | --- | --- | --- | --- |
|  |  |  | x | y | z |  |  |  |
| undefined |  | R | 17 | 15 | -3 | 38241 | 19.47 | <0.001 |
|  | undefined | R | 24 | 11 | -12 | s.c | 16.91 | <0.001 |
|  | Putamen | R | 26 | 18 | -6 | s.c | 16.66 | <0.001 |
| Superior Frontal |  | R | 20 | 65 | 12 | 1259 | 6.17 | <0.001 |
|  | Superior Frontal Medial | R | 6 | 50 | 6 | s.c | 5.88 | <0.001 |
|  | Superior orbital frontal | R | 27 | 62 | -2 | s.c | 5.76 | <0.001 |
| Superior Frontal |  | R | 21 | 36 | 51 | 155 | 6.05 | <0.001 |
|  | Middle Frontal | R | 27 | 23 | 54 | s.c | 4.98 | <0.001 |
|  | Superior Frontal | R | 21 | 29 | 57 | s.c | 4.04 | <0.001 |
| Superior Occipital |  | L | -15 | -85 | 28 | 207 | 5.46 | <0.001 |
|  | Cuneus | L | -11 | -87 | 15 | s.c | 4.18 | <0.001 |
| Precuneus |  | R | 8 | -52 | 6 | 110 | 5.26 | <0.001 |
| undefined |  | R | 2 | -39 | -24 | 202 | 5.17 | <0.001 |
| Lingual |  | L | -12 | -76 | 0 | 234 | 5.16 | <0.001 |
|  | Lingual | L | -17 | -67 | -2 | s.c | 4.7 | <0.001 |
| Paracentral Lobule |  | R | 12 | -33 | 58 | 353 | 5.12 | <0.001 |
|  | undefined | R | 15 | -24 | 61 | s.c | 4.96 | <0.001 |
|  | Paracentral Lobule | R | 12 | -24 | 69 | s.c | 4.14 | <0.001 |
| Inferior Parietal |  | L | -57 | -54 | 37 | 512 | 5.08 | <0.001 |
|  | Middle Temporal | L | -62 | -52 | 12 | s.c | 4.95 | <0.001 |
|  | Middle Temporal | L | -65 | -43 | 3 | s.c | 4.35 | <0.001 |
| Thalamus |  | L | -8 | -18 | 0 | 257 | 4.98 | <0.001 |
|  | Thalamus | L | -18 | -27 | 7 | s.c | 4.34 | <0.001 |
|  | Thalamus | L | -14 | -28 | -2 | s.c | 3.97 | <0.001 |
| Fusiform |  | R | 29 | -82 | -3 | 205 | 4.84 | <0.001 |
|  | Lingual | R | 17 | -82 | 0 | s.c | 4.75 | <0.001 |
|  | Lingual | R | 15 | -72 | 3 | s.c | 4.7 | <0.001 |

Peak regions are within the Main cluster

Max T is the maximum T statistic for each local maximum. FDR P<0.0001 based on non-stationary cluster-extent False discovery rate correction. s.c: same clusters
