# Supplementary material for: Catechol-O-Methyltransferase Val158Met Polymorphism on Striatum Structural Covariance Networks in Alzheimer’s Disease
Source: Mol Neurobiol. 2017 Jul 13;55(6):4637–49. doi: 10.1007/s12035-017-0668-2 (PMC5948254; doi:10.1007/s12035-017-0668-2)
Supplement: Supplementary file 23 — (DOCX 23 kb) [file 12035_2017_668_MOESM22_ESM.docx]

**Supplementary table 21. Covariances interactions of two catechol-O-methyltransferase genotype groups**

| **Seed Regions** | **Peak regions** | **Side** | **Stereotaxic coordinates** | | | | | **Extent** | | **MaxT** | **P-value** |
| --- | --- | --- | --- | --- | --- | --- | --- | --- | --- | --- | --- |
|  |  |  | x | y | | z | |  | |  |  |
| Met carrier> Valine homozygotes in the triple network model | | | | | | | | | | | |
| Entorhinal seed | Superior medial Frontal | L | -8 | 47 | | 34 | | 256 | | 3.92 | <0.001 |
| Met carrier> Valine homozygotes in the Basal ganglia network | | | | | | | | | | | |
| VSs seed | Caudate | L | -15 | | -3 | | 24 | | 143 | 3.72 | <0.001 |
| DC seed | Caudate | L | -18 | | -18 | | 21 | | 355 | 4.23 | <0.001 |
|  | Caudate | R | 18 | | -1 | | 22 | | 238 | 4.08 | <0.001 |
| DCP seed | Superior Occipital | R | 32 | | -64 | | 40 | | 351 | 4.59 | <0.001 |
|  | Middle Occipital | L | -27 | | -75 | | 34 | | 530 | 4.43 | <0.001 |
|  | Superior Parietal | L | -24 | | -60 | | 46 | | 157 | 4.21 | <0.001 |
|  | Postcentral | L | -20 | | -31 | | 60 | | 174 | 3.92 | <0.001 |
|  | Caudate | L | -15 | | -1 | | 22 | | 156 | 3.58 | <0.001 |
| DRP seed | Angular | R | 32 | | -63 | | 42 | | 620 | 5.19 | <0.001 |
|  | Middle Occipital | L | -27 | | -73 | | 34 | | 1211 | 4.87 | <0.001 |
|  | Paracentral Lobule | R | 2 | | -24 | | 70 | | 147 | 4.39 | <0.001 |
|  | Caudate | L | -15 | | -1 | | 22 | | 301 | 4.2 | <0.001 |
|  | Postcentral | L | -21 | | -30 | | 58 | | 140 | 3.97 | <0.001 |
|  | Supplementary Motor Area | L | -6 | | 0 | | 67 | | 116 | 3.72 | <0.001 |
|  | Postcentral | R | 24 | | -30 | | 58 | | 113 | 3.57 | <0.001 |
| VRP seed | Middle Occipital | L | -27 | | -72 | | 34 | | 1024 | 5.02 | <0.001 |
|  | Angular | R | 30 | | -63 | | 40 | | 471 | 4.99 | <0.001 |
|  | Precentral | L | -24 | | -28 | | 55 | | 331 | 4.71 | <0.001 |
|  | Caudate | L | -18 | | -19 | | 22 | | 338 | 4.63 | <0.001 |
|  | Superior Frontal | R | 26 | | 0 | | 54 | | 166 | 4.31 | <0.001 |
|  | Putamen | R | 29 | | 12 | | 1 | | 154 | 4.19 | <0.001 |
|  | Precuneus | R | 6 | | -69 | | 48 | | 684 | 4.11 | <0.001 |
|  | Precuneus | L | -6 | | -63 | | 49 | | 173 | 4.07 | <0.001 |
|  | Supplementary Motor Area | L | -6 | | 0 | | 66 | | 156 | 3.96 | <0.001 |
|  | Inferior Parietal | L | -42 | | -37 | | 42 | | 108 | 3.75 | <0.001 |

Peak regions are within the Main cluster; R=right, L=left;

Max T is the maximum T statistic for each local maximum. P<0.001with cluster size=100

PCC=Posterior cingulate cortex dorsal caudate (DC), ventral caudate superior (VSs), ventral caudate/nucleus accumbens inferior(VSi), dorsal rostral putamen (DRP), dorsal caudal putamen (DCP),
