# Supplementary material for: Catechol-O-Methyltransferase Val158Met Polymorphism on Striatum Structural Covariance Networks in Alzheimer’s Disease
Source: Mol Neurobiol. 2017 Jul 13;55(6):4637–49. doi: 10.1007/s12035-017-0668-2 (PMC5948254; doi:10.1007/s12035-017-0668-2)
Supplement: Supplementary file 24 — (DOCX 25 kb) [file 12035_2017_668_MOESM23_ESM.docx]

**Supplementary Table 22.** **Correlation matrix between cognitive test scores and seed volume in the triple network model in each catechol-O-methyltransferase genotype group**

| Seed # | Right Entorhinal | |  | Left Posterior Cingulate | |  | Right Frontoinsular | | Right Dorsolateral prefrontal | | | |
| --- | --- | --- | --- | --- | --- | --- | --- | --- | --- | --- | --- | --- |
| Genotype groups | Met-carriers | Val/Val |  | Met-carriers | Val/Val |  | Met-carriers | Val/Val |  | Met-carriers | Val/Val |  |
| Minimental state examination | 0.08 | 0.09 |  | **0.29**** | **0.26**** |  | 0.21 | 0.11 |  | 0.09 | 0.08 |  |
| CASI total scores | 0.04 | 0.08 |  | **0.31**** | **0.24**** |  | 0.13 | 0.07 |  | 0.16 | 0.05 |  |
| Executive function test | 0.04 | 0.08 |  | **0.31**** | **0.24**** |  | 0.13 | 0.06 |  | 0.15 | 0.06 |  |
| Mental Manipulation | -0.54 | 0.08 |  | **0.16** | **0.27**** |  | -0.03 | 0.04 |  | 0.04 | -0.05 |  |
| Attention | 0.02 | 0.04 |  | **0.31**** | **0.23**** |  | 0.16 | 0.11 |  | -0.06 | 0.07 |  |
| Orientation | 0.07 | 0.11 |  | **0.30**** | 0.17 |  | 0.16 | 0.04 |  | 0.15 | 0.00 |  |
| Long term memory | 0.05 | 0.08 |  | **0.29**** | 0.09 |  | 0.12 | -0.04 |  | 0.15 | -0.05 |  |
| Short term memory | 0.12 | -0.01 |  | **0.27**** | 0.19 |  | 0.18 | 0.14 |  | 0.16 | 0.05 |  |
| Abstract thinking | 0.06 | 0.08 |  | **0.26**** | 0.15 |  | 0.05 | 0.07 |  | **0.23*** | 0.13 |  |
| Drawing | 0.05 | 0.03 |  | **0.08** | 0.19 |  | 0.06 | -0.07 |  | 0.10 | 0.06 |  |
| Verbal fluency | -0.16 | 0.05 |  | **0.24**** | 0.18 |  | 0.10 | 0.08 |  | 0.19 | 0.15 |  |
| Language | 0.04 | 0.10 |  | **0.33**** | **0.26**** |  | 0.16 | 0.10 |  | -0.02 | 0.15 |  |
| NPI total scores | 0.01 | 0.15 |  | -0.03 | -0.10 |  | 0.10 | -0.01 |  | -0.06 | -0.19 |  |
| Delusion | 0.00 | 0.09 |  | -0.10 | -0.07 |  | 0.00 | 0.08 |  | -0.06 | -0.05 |  |
| Hallucination | 0.11 | -0.11 |  | -0.04 | 0.01 |  | 0.02 | -0.15 |  | -0.05 | -0.11 |  |
| Aggression | -0.13 | 0.12 |  | 0.00 | 0.02 |  | -0.07 | 0.04 |  | -0.18 | -0.02 |  |
| Depression | 0.00 | 0.10 |  | -0.09 | -0.15 |  | 0.11 | 0.18 |  | -0.13 | -0.04 |  |
| Anxiety | 0.03 | 0.14 |  | -0.05 | -0.02 |  | 0.03 | 0.14 |  | -0.02 | -0.06 |  |
| Elation | -0.09 | 0.01 |  | -0.07 | **-0.29**** |  | -0.14 | 0.00 |  | **-0.23**** | -0.14 |  |
| Apathy | -0.09 | 0.00 |  | 0.02 | -0.13 |  | -0.07 | -0.10 |  | -0.07 | -0.03 |  |
| Disinhibition | 0.00 | 0.13 |  | -0.04 | -0.11 |  | 0.05 | -0.07 |  | 0.01 | **-0.22**** |  |
| Irritability | 0.07 | 0.10 |  | 0.08 | 0.02 |  | 0.10 | 0.15 |  | 0.05 | -0.13 |  |
| Aberrant motor behavior | -0.06 | 0.04 |  | 0.12 | -0.14 |  | -0.12 | -0.10 |  | -0.09 | -0.08 |  |
| Sleep disorder | 0.06 | 0.09 |  | -0.05 | 0.07 |  | **0.28**** | -0.15 |  | 0.14 | -0.11 |  |
| Eating behavior | 0.13 | -0.07 |  | -0.02 | 0.01 |  | 0.00 | -0.04 |  | -0.11 | -0.14 |  |

# The 4 seeds represented Default mode medial temporal lobe subsystem and midline core subsystem, Salience network and Executive control network

Numbers indicate Pearson correlation coefficients, *p<0.05; ** <0.01

CASI= cognitive ability screening instrument; NPI= Neuropsychiatric inventory
